# Physical simulation for water invasion and water control optimization in water drive gas reservoirs

**DOI:** 10.1038/s41598-021-85548-0

**Published:** 2021-03-18

**Authors:** Xuan Xu, Xizhe Li, Yong Hu, Qingyan Mei, Yu Shi, Chunyan Jiao

**Affiliations:** 1 PetroChina Research Institute of Petroleum Exploration and Development, Beijing, 100083 China; 2 Research Institute of Southwest Oil and Gas Field Company, Sichuan, 610051 China; 3 Xi’an Shiyou University, Shanxi, 710065 China

**Keywords:** Energy science and technology, Engineering, Physics

## Abstract

The development of water drive gas reservoirs (WDGRs) with fractures or strong heterogeneity is severely influenced by water invasion. Accurately simulating the rules of water invasion and drainage gas recovery countermeasures in fractured WDGRs, thereby revealing the mechanism of water invasion and an appropriate development strategy, is important for formulating water management measures and enhancing the recovery of gas reservoirs. In this work, physical simulation methods were proposed to gain a better understanding of water invasion and to optimize the water control of fractured WDGRs. Five groups of experiments were designed and conducted to probe the impacts of the distance between the fractures and the gas well, the drainage position, the drainage timing and the aquifer size on the water invasion and production performance of a gas reservoir. The gas and water production and the internal pressure drop were monitored in real time during the experiments. Based on the above experimental works, a theoretical analysis was conducted to quantitatively evaluate the performance of the gas reservoir recovery via the gas well production performance, water invasion, dynamic pressure drop and residual gas and water distribution analysis. The results show that when the fracture scale was appropriate, a gas well drilled close to a fracture (Experiment 1-3) or a high-permeability formation could also produce gas and achieve drainage efficiently. The recovery factor of Experiment 1-3 reached 62.5%, which was 24.6% and 21.1% higher than those of Experiments 1-1 and 1-2, respectively, which had wells drilled in low-permeability areas. Draining water near an aquifer can effectively inhibit water invasion during the early stage of gas recovery. The setup in Experiment 2-1 effectively inhibited water invasion and avoided the formation of water-sealed volumes of gas to recover 30% more gas than recovered with that of Experiment 1-1 without drainage wells. A shorter distance between the drainage well and the aquifer increased the drainage capacity and decreased the gas production capacity, respectively (Well 2 at Point A vs Point B). A larger aquifer had a lower gas recovery, which reduced the economic benefit. For example, due to an infinitely large aquifer, the reserves in Experiment 4-1 were developed by a single well, the gas recovery was only 33.4%. These research results are expected to be beneficial for the preparation of development plans and the optimization of water control measures for WDGRs.

## Introduction

Most of the gas reservoirs discovered and developed in China are affected by water invasion, albeit to different degrees. Such effects have been observed to be more serious for WDGRs with fractured or highly heterogeneous formations. The edge water or bottom water tends to intrude easily through fractures or high-permeability zones during gas production. This splits the gas reservoir, resulting in water production in the gas wells, which considerably decreases gas production^1–4^.

WDGRs have the advantage of supplemented energy. For a relatively homogeneous reservoir, the water intrudes evenly, forming the so-called tongue, which acts to replenish energy^5,6^. However, water intrusion poses a larger risk to gas reservoir development than to oil reservoir development. The edge and bottom water will "channel in" through the high-permeability zones in a fractured or heterogeneous reservoir, decreasing the efficiency of gas reservoir development. After the water in the gas reservoir is discharged, the formation water intrudes through the gas reservoir and will be split due to the formation water invasion via the fractures (or high-permeability zones). Then, some gas zones will be sealed and eventually form a dead zone, resulting in a significant decrease in the ultimate recovery of the gas reservoir. Additionally, after the gas wells are discharged, gas and water two-phase flow occurs in the formation, which will lead to an increased gas reservoir abandonment pressure and decreased gas production. In the later stage, due to the need for drainage to stabilize the production and tap potential, the difficulty and the costs of development increase^7^.

Numerous efforts have been made regarding gas reservoir water invasion control by researchers in the oil and gas industry. There are currently three main types of water control countermeasures, i.e., water drainage, gas well deliquification, and water shut-off ^8–10^. Water shut-off is performed to reduce the permeability of fractures by injecting chemical agents, such as gelant, into them, thus preventing water invasion. Ghosh et al. presented experimental results of water shut-off and noted that the reservoir matrix must be well protected while plugging fractures^11^. To achieve this, different agents may need to be injected into the reservoir at different times. This study suggested that it is not easy to solve the problem of water invasion via water shut-off. Among the three types of water control countermeasures, water drainage uses water wells to actively produce water to consume water energy and reduce the pressure difference between the gas zones and water zones to control water invasion. Water drainage, especially a multiwell combined drainage scheme implemented across a gas reservoir, is an active water control strategy that is widely used in practice. However, due to the complex reservoir conditions and water invasion mechanisms, as well as the constraints imposed by the technical, economic, and environmental conditions, it is difficult to determine the best water control measures and timing, posing great challenges to the formulation and implementation of water control development in gas reservoirs^12^.

Therefore, for formulating water management measures and enhancing the recovery ratio of gas reservoirs, it is important to accurately simulate the water invasion and drainage gas recovery countermeasures in fractured WDGRs to reveal the water invasion mechanism and plan the reservoir development strategy^13–17^. However, due to the complexity of the water invasion mechanism and the limitations of experimental techniques, there have been few targeted theoretical studies performed on water control in such a WDGR. Lakatos et al. illustrated the water-induced formation damage via a flow experiment and high-pressure Hg porosimetry of tight sandstone^18^. Li and Zhang conducted an experimental study of water shut-off by gas wettability alteration and investigated the feasibility of reducing water production in gas wells by changing the wettability of the gas zone^19^. Xu et al. investigated the water drainage effect of reducing water invasion via physical simulation experiments^20^. Fang et al. investigated the influence of reservoir parameters on water invasion in fractured carbonate gas reservoirs^21^. A few indoor studies were conducted on the entire development cycle of gas reservoirs, including water invasion in the early stages and water control in the later stages^22,23^.

In this work, a typical gas reservoir is adapted as a prototype to establish a geological model considering different fracture and matrix parameters. Such a model is aimed at simulating the relationship between the fractures and gas well locations, the water invasion in fractured WDGRs with different drainage locations, the drainage timing and the water body sizes. The pressure profiles of different gas reservoirs are monitored and recorded during the experiments to reveal the water invasion dynamics, i.e., the water sealing mechanism. Accordingly, reserve development rules and the distribution of the residual gas are analyzed. On these bases, reasonable countermeasures are proposed, providing technical support for the preparation of development plans and water control development measures for this type of gas reservoir.

## Experiments

### Geological model

**Figure 1:**
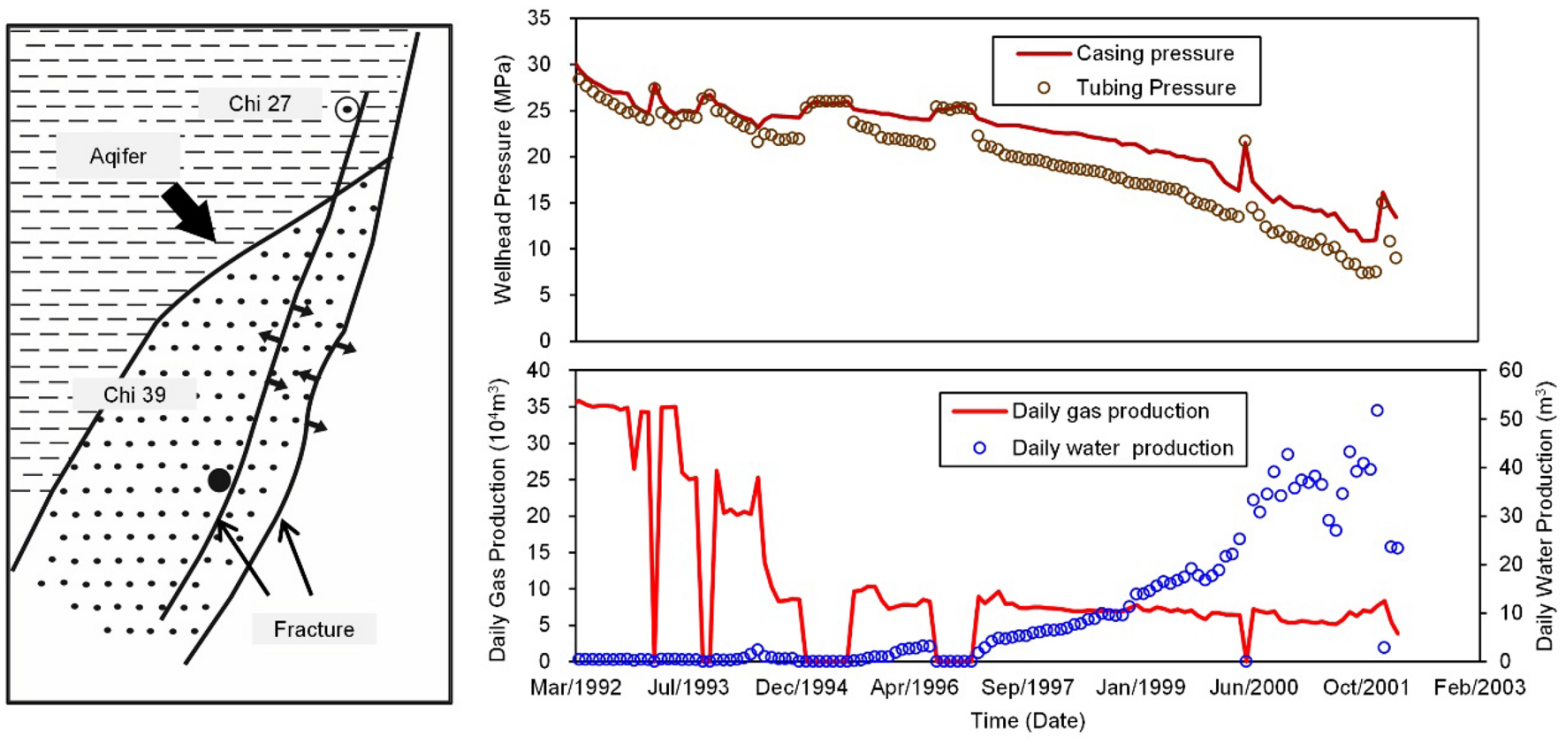
Schematic diagram and production history of Chi 27-39 well in the gas field in eastern Sichuan, China. Modified according to Gou et al.^27^.

The water control practices in the Longdiao gas reservoir, a Carboniferous gas field in eastern Sichuan, China, reflect the complexity and challenges of on-site water control^24,25^. The porosity of Longdiao gas reservoir is generally 1–19%, with an average of 5.4%; the permeability span of the reservoir is large, generally 0.001md–10.0mD, with an average of 4.0mD; The permeability of fractured reservoir is generally 10–100mD, according to the researchers^26,27^. Well Chi 39 in the Longdiao gas reservoir is located at the northern end of the Diaozhongba high spot of the gas field, with single-well-controlled reserves of 26.5 × 10^8^ m^3^. This well was put into production in March 1992. The initial daily gas production rate (${\text{Q}}_{{\text{g}}}$) was 35.0 × 10^4^ m^3^/d. In March 1994, formation water was suddenly produced at a daily water production rate (${\text{Q}}_{{\text{w}}}$) of 3 m^3^/d. The daily ${\text{Q}}_{{\text{g}}}$ was then reduced to 7.3 × 10^4^ m^3^/d. Studies showed that Well Chi 39 underwent water invasion typical of reservoirs with large fractures: two large water-conducting faults bring water to the Well Chi 39 area, as shown in Fig. 1.

In this case, the water invasion control measures for the gas reservoir were as follows:

In May 1998, a large-scale water drainage for pressure relief was performed for Well Chi 27; at the same time, gas lift drainage was conducted for Well Chi 39 using two additional wells for water control. The dynamic monitoring curves of the gas production depicted that the pressure in the water zones of Well Chi 27 was obviously decreased due to the pumping and drainage, and the daily ${\text{Q}}_{{\text{w}}}$ of Well Chi 39 was effectively stabilized. After pumping was stopped in October 1999, the pressure in the water zones around Well Chi 27 rapidly recovered. The formation filtration conditions at Well Chi 39 deteriorated rapidly, with the rapid decrease in casing pressure. The ${\text{Q}}_{{\text{w}}}$ increased from 18 m^3^/d at pumping stoppage to approximately 40 m^3^/d. The positive and negative production changes indicated that the pumping drainage pressure relief had a significant effect on inhibiting the further deterioration of water invasion and reducing the water invasion damage. Although there was a favorable trend in the initial stage of drainage, after nearly four years of drainage, the overall water control effect in the area of Well Chi 27 has not achieved its intended purpose. The main reason is that the water displacement from Well Chi 27 did not meet the designed requirement, and the energy of the movable water was greater than predicted. The water control practice applied in the Well Chi 27-39 area shows that the uncertainties of the water-energy prediction and the actual drainage conditions on site are important factors affecting the water control effectiveness in the well area.

**Figure 2:**
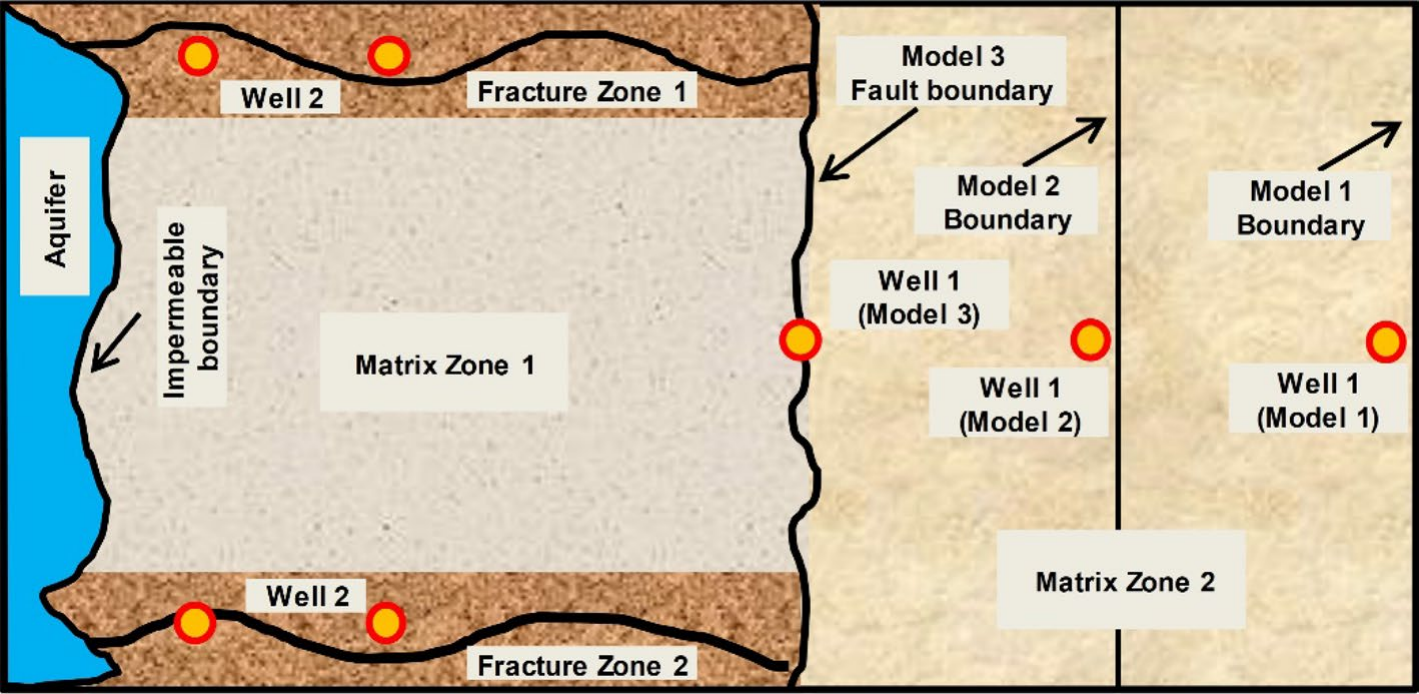
Multi-well joint water-control geological model for fractured water drive gas reservoirs (three geological models).

The Chi 27-39 well area of the Longdiao gas reservoir mentioned above has the typical characteristics of a WDGR. As shown in Fig. 2, three geological models of typical multiscale fractured WDGR are developed, taking the geological and development examples of the gas zone as a prototype. Water invasion and water control experiments are designed and performed. The left end of the model is connected to the aquifer, and the right side is a heterogeneous reservoir, which is composed of fractured zones (FZs) and matrix zones (MZs) with different physical properties. The FZs are located above and below the MZs. The MZs are divided into MZ 1 and MZ 2 according to different physical properties (indicated by the different filling colors in Fig. 2). The main differences among the three geological models are the range of the right MZ 2 (see the boundaries of the three models shown in Fig. 2) and the location of Well 1. The gas wells in Geological Models 1 and 2 were deployed at MZ 2, representing a gas reservoir with low-permeability matrix barriers; in these cases, the gas wells and aquifer are not completely connected via fractures. In Model 3, Well 1 is directly connected to the FZ, which represents a gas reservoir in which the gas wells are directly connected with the aquifer through fractures. Compared to the other models, in Model 1, Well 1 is the farthest from the FZ; in Model 3, Well 1 is directly connected to the FZ.

### Experimental method

**Figure 3:**
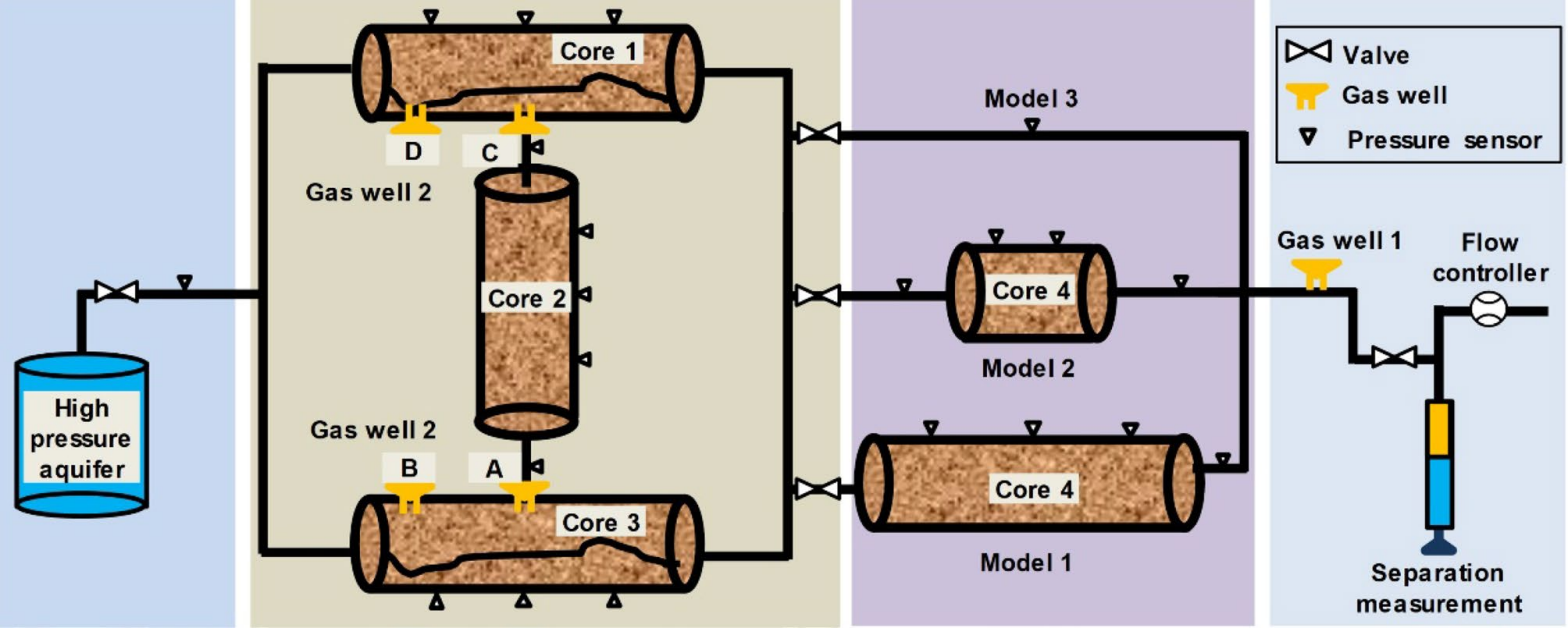
Schematic diagram of the joint water-control experiments (three geological models, Well 2 at points A/B/C/D).

**Figure 4:**
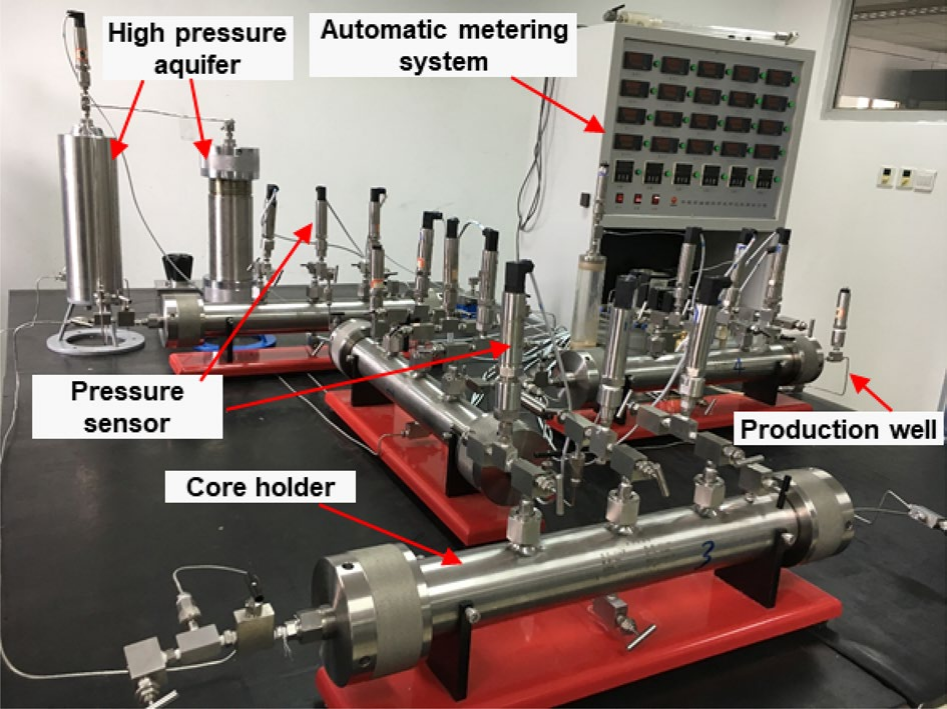
Joint water-control experimental setup for fractured water drive gas reservoirs.

Corresponding to the geological model, a novel experimental device and method for water invasion and water control in a complex and fractured WDGR were proposed and established (Figs. 3 and 4).

As shown in Figs. 3, 4 and Table 1, the experimental reservoir was mainly composed of four groups of cores with different physical properties. The core holders were equipped with multiple pressure probes to monitor the gas reservoir pressure profile in real time.

**Table 1:** Basic physical parameters of the four groups of cores used in the experiments.

Core No	Reservoir types	Length, cm	Diameter, cm	Porosity, %	Permeability, mD
1	Through-going fracture	50	2.52	5.42	9.68
2	Pore type	50	2.51	4.54	0.67
3	Through-going fracture	50	2.53	5.63	18.08
4	Pore type	50/25/0	2.51	5.91	2.54

Core 1 and Core 3, which were fractured artificially, were used to represent the small-fracture zone (SFZ) and the large-fracture zone (LFZ), respectively, while Core 2 and Core 4 represent the MZs.

Different lengths were selected for Core 4 according to the simulated geological models: 50 cm for Model 1; 25 cm for Model 2; and 0 cm for Model 3. For Well 1 in the three models, the fracture penetration rates were 50%, 67%, and 100%, respectively (Table 1).

Five groups of different types of experiments were designed to investigate the main geological and production conditions affecting the water invasion and water control in the gas reservoirs. The purpose, characteristics, models, and gas well parameters of the experiments are shown in Table 2 and are described in detail below:

**Table 2:** Contents and parameters of different groups of experiments.

Group No	Experimental No	Factors	Experimental characteristics	Location of Gas Well 2	Geological model	Aquifer
I	1-1	Location relationship between gas wells and fracture zones	Single well production in Gas Well 1 The distance from Well 1 to the fractured zones are different	n/a	Model 1	Finite aquifer (15 times)
	1-2			n/a	Model 2	
	1-3			n/a	Model 3	
II	2-1	Drainage position	Gas Well 1 and 2 producing at the same time Well 2 is set at a different position of the fractured zone	Middle of large fracture-A	Model 1	Finite aquifer (15 times)
	2-2			Near water end of large fracture-B	Model 1	
	2-3			Middle of small fracture-C	Model 1	
	2-4			Near water end of small fracture-D	Model 1	
III	3-1	Drainage timing	Well 2 was started up immediately after production was stopped in Well 1	Middle of large fracture-A	Model 2	Finite aquifer (15 times)
	3-2		Well 2 was started up in 16 h after production in Well 1 was stopped	Middle of large fracture-A	Model 2	
	3-3		Well 2 was started immediately after production was stopped in Well 1	Middle of large fracture-A	Model 3	
IV	4-1	Aquifer size	Well 2 was started immediately after production was stopped in Well 1 Different Aquifer	Middle of large fracture-A	Model 2	Infinite aquifer
	4-2			n/a	Model 2	n/a
V	5-1	Basic experiment	No aquifer, only Well 1 was producing	n/a	Model 1	n/a
	5-2			n/a	Model 3	n/a

The first group of experiments (Group I) was designed to unveil the impact of the positional relationship between the gas wells and FZs on the water invasion. During the single-well production of Well 1, three different geological models were formed by adjusting the length of Core 4, as described above.

The second group of experiments (Group II) was designed to reveal the impact of the drainage positions on the water control effectiveness. Wells 1 and 2 were simultaneously initiated for production, simulating multiple-well drainage and gas production. Note that Well 2 was placed at four different positions (A, B, C, and D) in the FZs in the four different experiments of Group II. A and C were set in the middle of Core 1 and Core 3, respectively. Meanwhile, B and D were 12.5 cm to the left of A and C, respectively.

The third group of experiments (Group III) was designed to conduct research related to the timing of drainage on water control effectiveness. Experiments 3-1 and 3-2 were completed with Geological Model 2, which was compared with Experiment 1-2 (considering the same geological model), and Experiment 3-3 was completed with Geological Model 3, which was compared with Experiment 1-3 (considering the same model).

The fourth group of experiments (Group IV) was designed to investigate the impact of aquifer size on water control effectiveness. Experiment 4-1 simulated gas reservoirs with an infinite aquifer, while Experiment 4-2 simulated gas reservoirs without an aquifer. Experiments 4-1 and 4-2 were compared with Experiment 3-1 because they used the same geological model. The volume of the aquifer in Experiment 3-1 was 15 times the gas reservoir volume.

The purpose of the fifth group of basic experiments (Group V) was a comparative analysis. Experiments 5-1 and 5-2 were conducted with Model 1 and Model 3, respectively, to simulate volumetric gas reservoirs.

In the first to third groups of experiments, the gas reservoirs were connected to the finite aquifer in a total of 10 experiments. Considering that the elastic energy was released from the gas reservoir rocks and the aquifer, an aquifer volume equivalent to 15 times the gas reservoir volume was consistently set in the experiments.

Considering the similar requirements and gas reservoir production matching experiences, the experimental production rate was 15–30% of the model open flow^28,29^. In this study, the measured open flows of the different models were in the range of 1000–3000 mL/min, so the experimental ${\text{Q}}_{{\text{g}}}$ met the open flow requirements of 150–900 mL/min. The abandoned production range of the gas reservoir and the detection accuracy of the flow meter was considered in the experimental study. The abandoned production was set to 2.5% of the ${\text{Q}}_{{\text{g}}}$. All the experimental gas wells were produced at a production rate of 400 mL/min with an abandonment production constraint of 10 mL/min.

### Experimental procedures

Step 1: Prepare the core models according to the experimental scheme, load all the cores into the core holder, and then add a confining pressure of 35 MPa.

Step 2: Slowly saturate the core model from both ends with nitrogen until the pressure reaches 30 MPa.

Step 3: Load the simulated formation water into a high-pressure resistant container and apply a pressure of 30 MPa. Change the aquifer setup as needed; if it is an infinite aquifer, then connect a constant pressure gas source to the container to provide continuous pressure.

Step 4: Connect the container storing the brine to the core model at 100% nitrogen saturation. According to the experimental scheme, produce the gas well at a production rate of 400 mL/min to simulate gas reservoir exploitation.

Step 5: During gas recovery, the pressure probes set on the core holder record the pressure profile of the core in real time. The instantaneous gas, water production, accumulated gas, water breakthrough time, and other parameters are recorded by the outlet flow meter and the gas–water separator. Stop the experiment when Well 1 or Well 2 reaches the predetermined production target.

Step 6: After the end of the experiment, weigh the cores of different positions to get the water saturation of the corresponding reservoir.

## Results and discussion

### Impact of the distance between the wells and fractures

In Group I, three different gas reservoir geological models were formed by varying the length of Core 4 to reveal the impact of the distance from the gas well to the fractures on the water invasion and gas production.

#### Production performance

**Figure 5:**
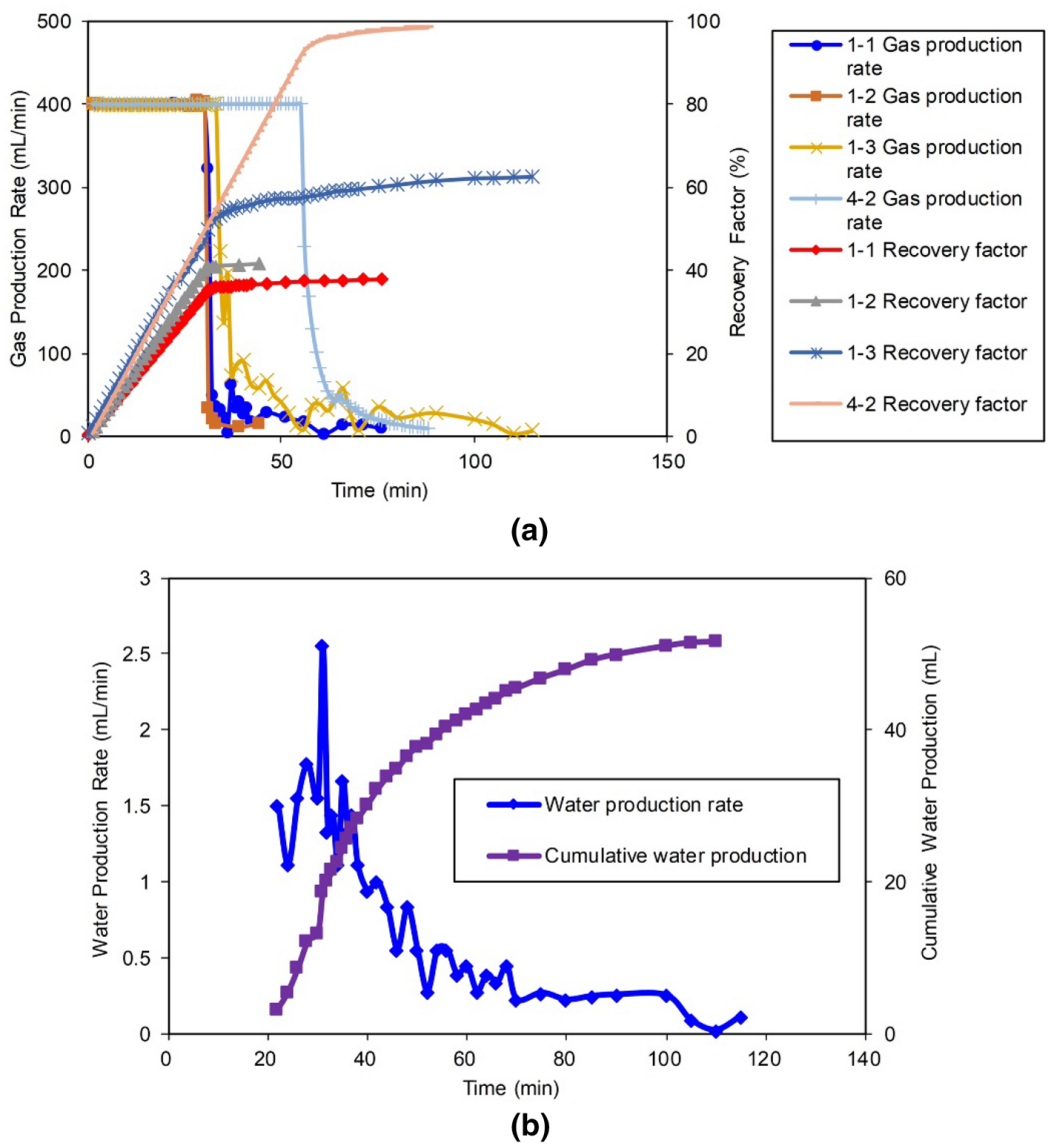
Production performance of the Group I: (**a**) gas production, and (**b**) water production of Experiment 1-3.

The production curves of Group I are plotted in Fig. 5. For comparison, the production curves of the volumetric gas reservoir in Experiment 4-2 are also included in Fig. 5. The main production parameters are given in Table 3.

**Table 3:** Statistics on the production of the Group I and Experiment 4-2.

Experimental No	Length of core 4, cm	Stable production period, min	${\text{R}}$ during stabilized period, %	Production period, min	${\text{R}}$, %	Breakthrough time, min	${\text{W}}_{{\text{p}}}$, ml
1-1	50	30	34.2	76	37.9	74	0.2
1-2	25	30	41.4	49	41.4	47	0.05
1-3	0	32	51.7	115	62.5	22	51.7
4-2	25	55	91.7	88	98.6	n/a	n/a

The experimental results show that the distance between the gas wells and the FZ can greatly influence the production performance and recovery factor (${\text{R}}$).

The gas wells in Gas Reservoirs 1-1 and 1-2 drilled in the low-permeability MZ 2, and there are barriers in the FZ. Once water breakthrough occurred in the gas wells in both gas reservoirs, the production rate dropped rapidly to the abandonment production rate. Specifically, water breakthrough was observed at 74 min in Experiment 1-1; then, at 76 min, the ${\text{Q}}_{{\text{g}}}$ decreased to the abandonment ${\text{Q}}_{{\text{g}}}$ due to the water breakthrough. Additionally, water breakthrough was observed at 47 min in Experiment 1-2; then, at 49 min, the gas production was stopped. Therefore, only the water production curves of Experiment 1-3 are included in Fig. 5b. For Experiment 1-3, with the gas well directly connected to the FZ, after water breakthrough, the gas well still produced gas for a long time. This is because the water in Well 1 could be quickly and fully drained during the experiment, although a water breakthrough was observed 22 min after Well 1 production was initiated, when the production capacity stabilized. Stable production lasted for 32 min, and both gas and water were produced for 115 min. The ${\text{R}}$ during the stage of producing both gas and water reached 25.6%.

Considering the timing of the water breakthrough, a shorter distance between a gas well and the FZ connected to an aquifer will result in a faster water breakthrough. The impacts of the water invasion and the water production capacities of the gas wells are varied, along with the different distances between the gas wells and the FZs. Although the ${\text{Q}}_{{\text{g}}}$ of the three types of gas reservoirs were similar, due to the large differences in the gas reservoir reserves, the ${\text{R}}$ of Experiment 1-1 was only 37.9%, and the ${\text{R}}$ of Experiment 1-2 was 41.4%, while the gas ${\text{R}}$ in Experiment 1-3 in the zone directly connected to the FZ reached 62.5%.

The experimental results show that because the FZ is connected to the aquifer, the closer a gas well is to the FZ, the higher the ${\text{ R}}$ is. First, the fractures have a high gas supply capacity because they are high-speed gas flow channels, which results in a stable production capacity of the gas reservoir; consequently, the reserves are developed fast. Second, the gas wells in the FZ can produce gas with water for a long time because continuous drainage consumes the water energy and inhibits the water invasion. Third, due to the high conductivity of the large fractures, even if the water saturation is increased, they still have a high seepage capacity, resulting in stabilized gas production at the gas wells after water breakthrough and maintaining long-term gas and water production. Notably, the conclusions have preconditions. The results on the effect of a fracture on the gas recovery are obtained from these experimental conditions (a specific fracture scale and matrix permeability). Fang et al. noted that although fractures at certain scales can enhance gas recovery, excessively large fractures would allow water to flow along the fractures, which could rapidly decrease the gas recovery^21^.

#### Water invasion analysis

Currently, the main methods of water invasion degree identification can be divided into three main methods: the pressure drop curve method, the apparent geological reserves method and the water invasion volume coefficient method^30–33^.

**Figure 6:**
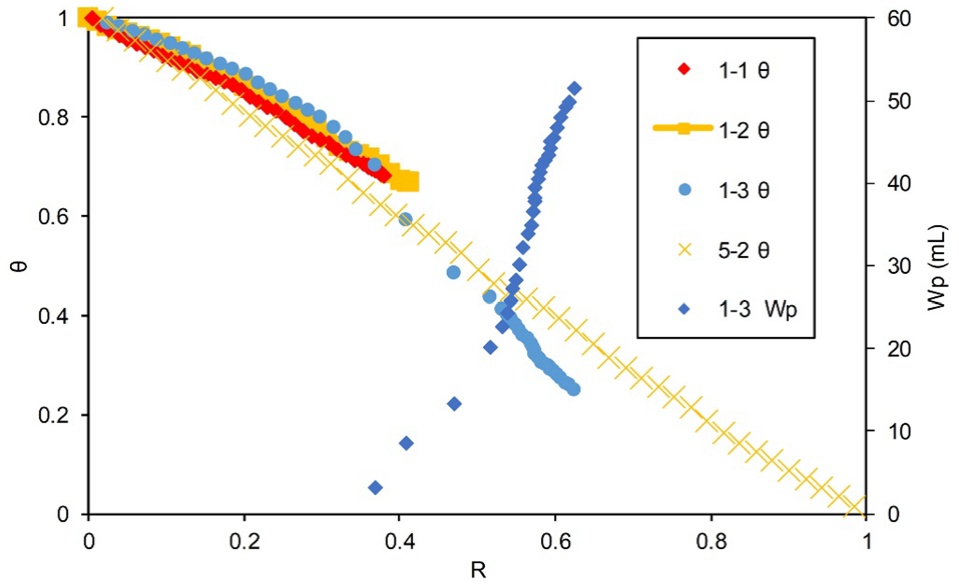
Relationship between relative apparent pressure of the formation and ${\text{R}}$.

Using the water invasion volume coefficient method, the $\uptheta \sim {\text{R}}$ curves of Group I were drawn; details are presented in Appendix A. For comparison, the curve of the basic Experiment 5-2 (curve of the volumetric gas reservoir Model 3 without an aquifer) was also drawn in Fig. 6. The relationships between the ${\text{W}}_{{\text{p}}}$ and the ${\text{R}}$ for Experiments 1, 2 and 3 are also plotted in Fig. 6 for ease of analysis.

Figure 6 shows a consistency among the theoretical results. For the volumetric gas reservoir depletion exploitation without an aquifer (Experiment 5-2), the $\uptheta \sim {\text{R}}$ curve basically conforms to the 45° line. In the early stage of production in Group I, the $\uptheta \sim {\text{R}}$ curve curves upward, which reflects the water invasion into the gas reservoirs. The $\uptheta \sim {\text{R}}$ curves of the later stage of production for the three experiments show a difference in the water invasion as follows.

1. For Experiments 1-1 and 1-2, the slopes of the $\uptheta \sim {\text{R}}$ curves do not change considerably, and they always plot above the 45° line, indicating that as the formation water continued to intrude, and effective drainage was not fulfilled. The gas well was directly connected to the FZ in Experiment 1-3. When *R* = 0.37 or so, the relative pressure of the drawdown curve begins to shift downward, showing strong drainage characteristics that correspond to the point where the gas well began to produce water. This indicates that the $\uptheta \sim {\text{R}}$ curve can, in time, accurately reflect the change in the water invasion degree on the basis of an accurate calculation of the average formation pressure and geological reserves. As the ${\text{W}}_{{\text{p}}}$ of the gas well increases, the relative pressure drawdown curve crosses the 45° line when the ${\text{R}}$ of the reserves *R* is approximately 0.4 and continues to tilt down, indicating that the net intrusion of the aquifer continued to decrease at this stage and gradually transformed into net production.
2. For Experiment 1-3, the $\uptheta \sim {\text{R}}$ curve decreases and finally approaches *θ* = 0.25, which is in accordance with the fact that the ${\text{R}}$ of Experiment 1-3 is more than 20% higher than those of Experiments 1-1 and 1-2.

#### Dynamic pressure drop

The gas reservoir material balance method can be adapted to estimate the overall water invasion degree and the ${\text{R}}$ of the gas reservoir. However, to calculate the ${\text{ R}}$ of the reservoir and the residual gas distribution in different zones of the gas reservoir, a deep understanding of the residual pressure of different regions of the gas reservoir is needed, and it is necessary to apply the gas reservoir pressure contour method and other methods to study the water sealing condition in the gas reservoir^34,35^. During the experiments, the dynamic pressure of the gas reservoir was monitored in real time to intuitively reflect the distribution of the residual sealed gas in the gas reservoir and the ${\text{R}}$ of the reserves, providing important analytical method and basis for understanding the water invasion rules, the water sealing gas mechanism and the mechanism of the remaining reserve development.

**Figure 7:**
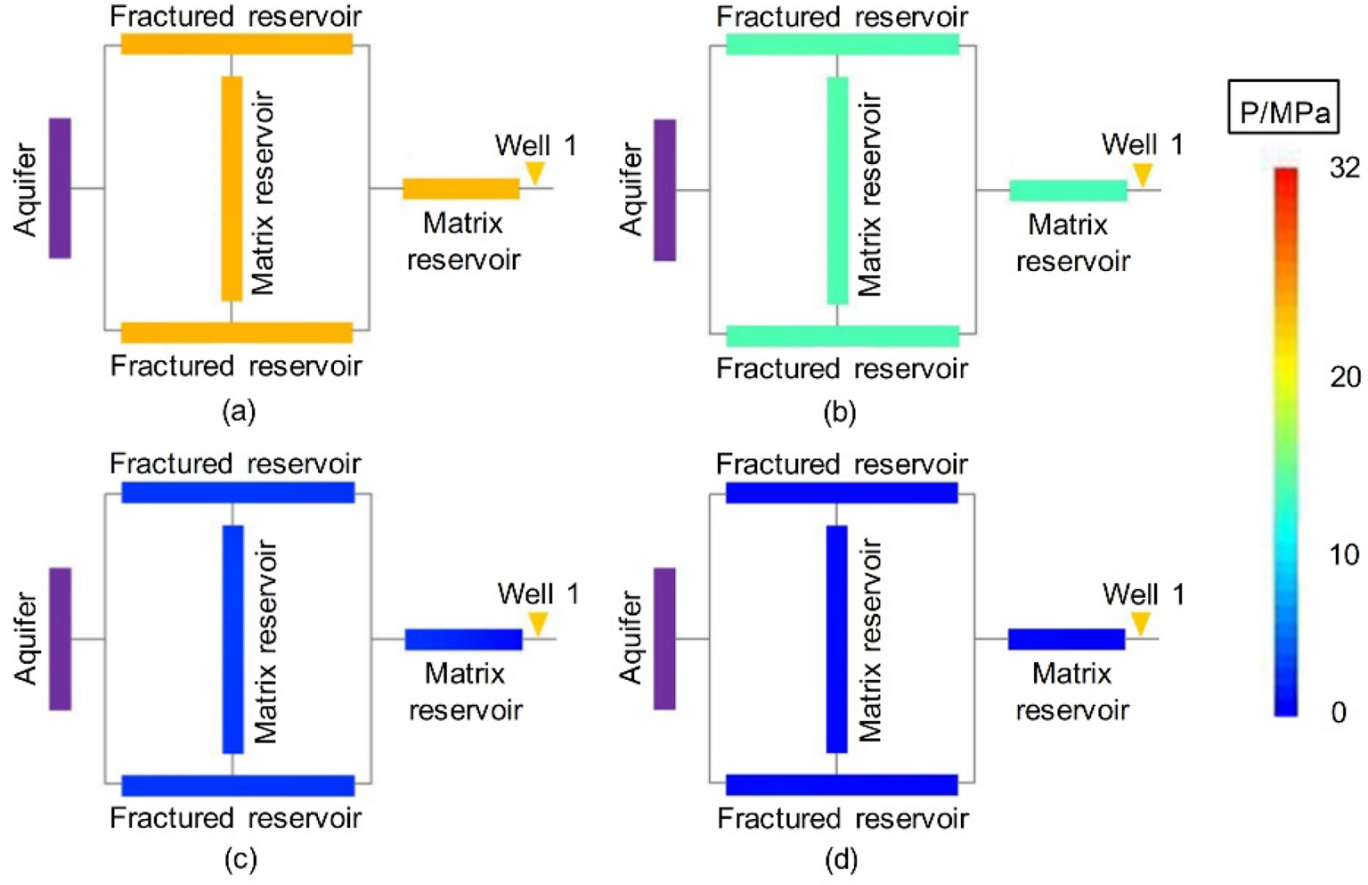
Pressure distributions at different stages of Experiment 4-2: (**a**) early stage of production – 10 min, (**b**) early stage of production—30 min, (**c**) end of the stabilized period—55 min, and (**d**) production halts—88 min.

**Figure 8:**
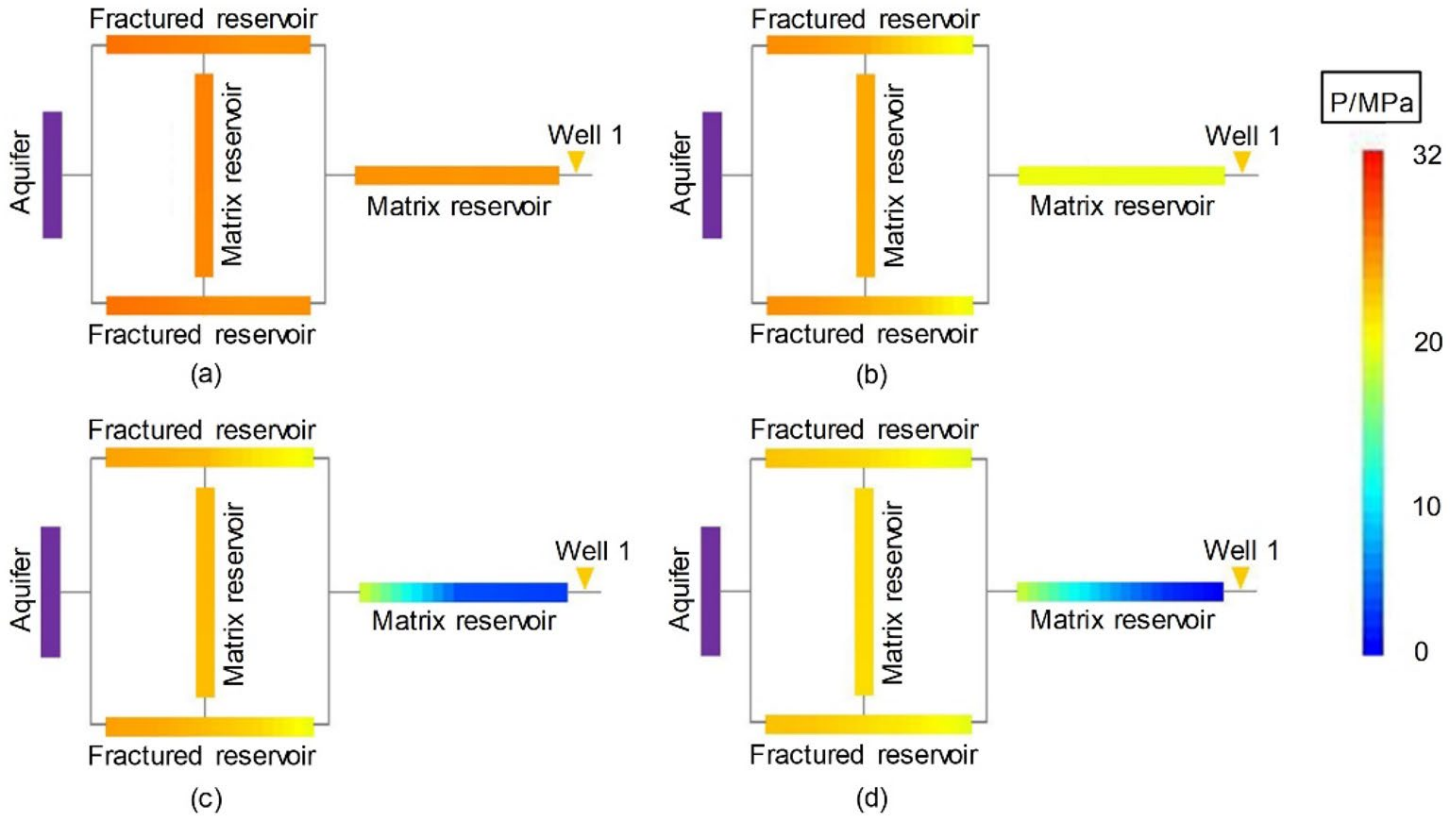
Pressure distributions at different stages of Experiment 1-1: (**a**) early stage of production—10 min, (**b**) early stage of production—20 min, (**c**) end of the stabilized period—30 min, and (**d**) production halts -76 min.

**Figure 9:**
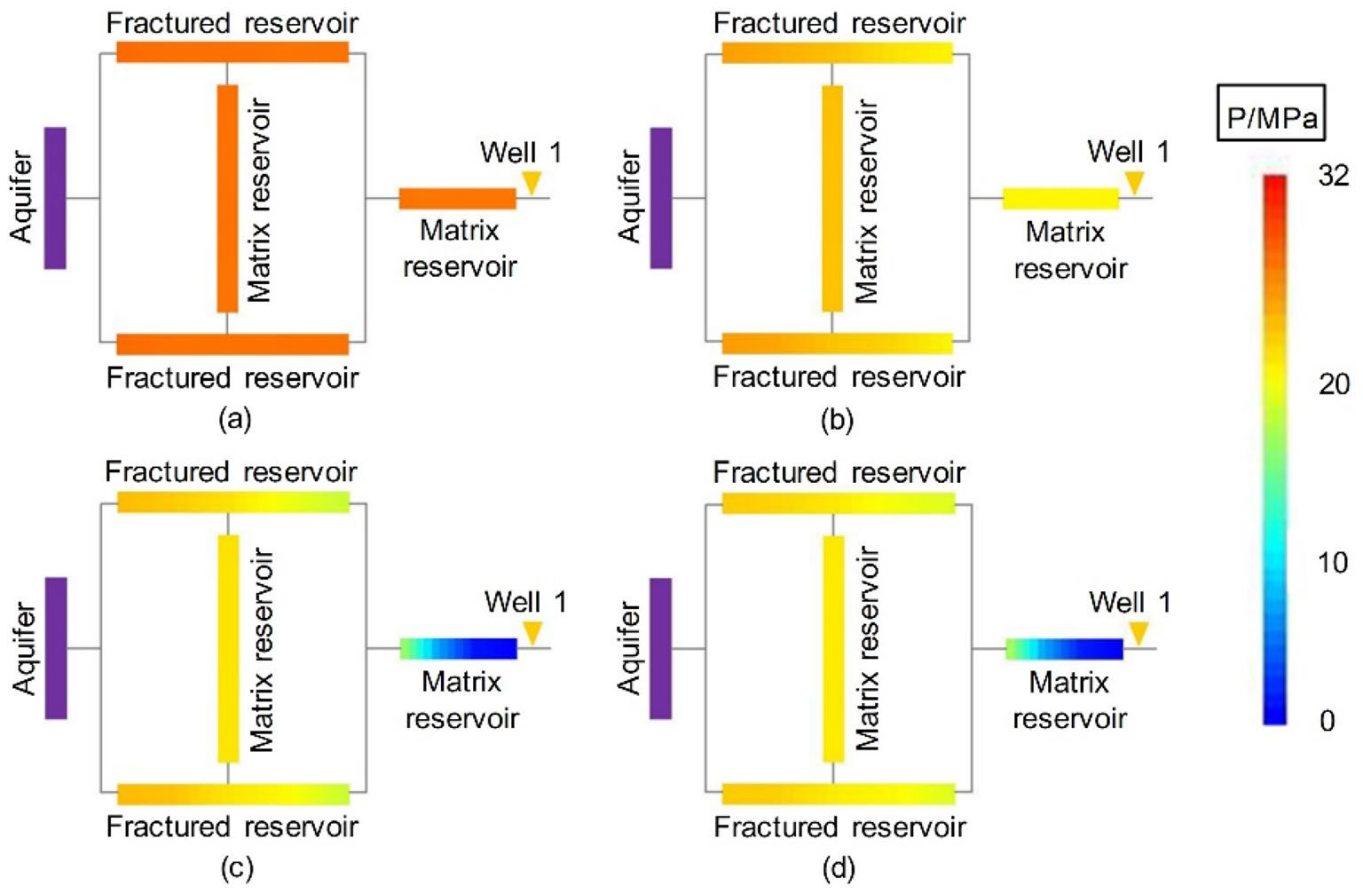
Pressure distributions at different stages of Experiment 1-2: (**a**) early stage of production—10 min, (**b**) early stage of production—20 min, (**c**) end of the stabilized period—30 min, and (**d**) production halts—49 min.

**Figure 10:**
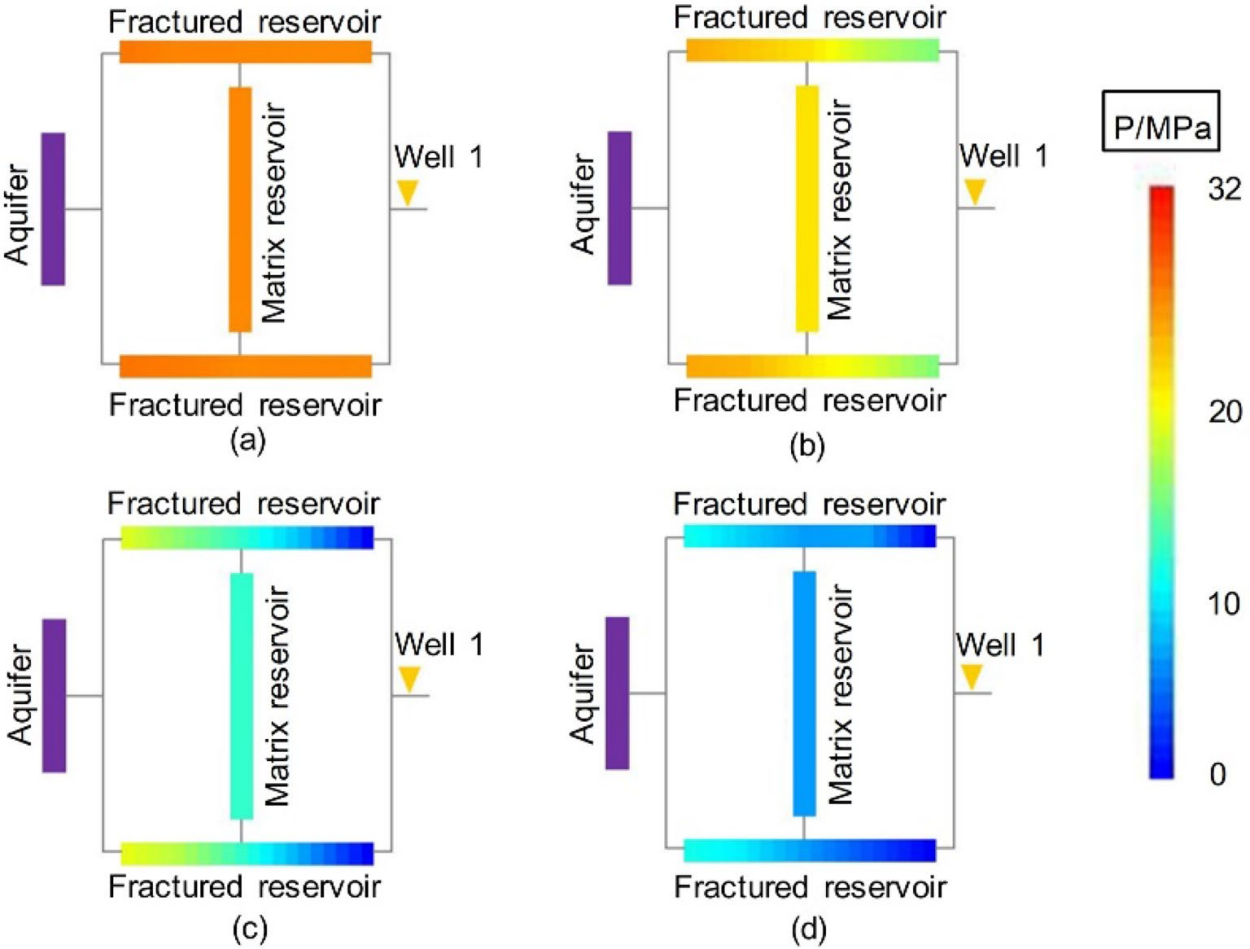
Pressure distributions at different stages of Experiment 1-3: (**a**) early stage of production—10 min, (**b**) early stage of production—20 min, (**c**) end of the stabilized period—32 min, and (**d**) production halts—115 min.

**Figure 11:**
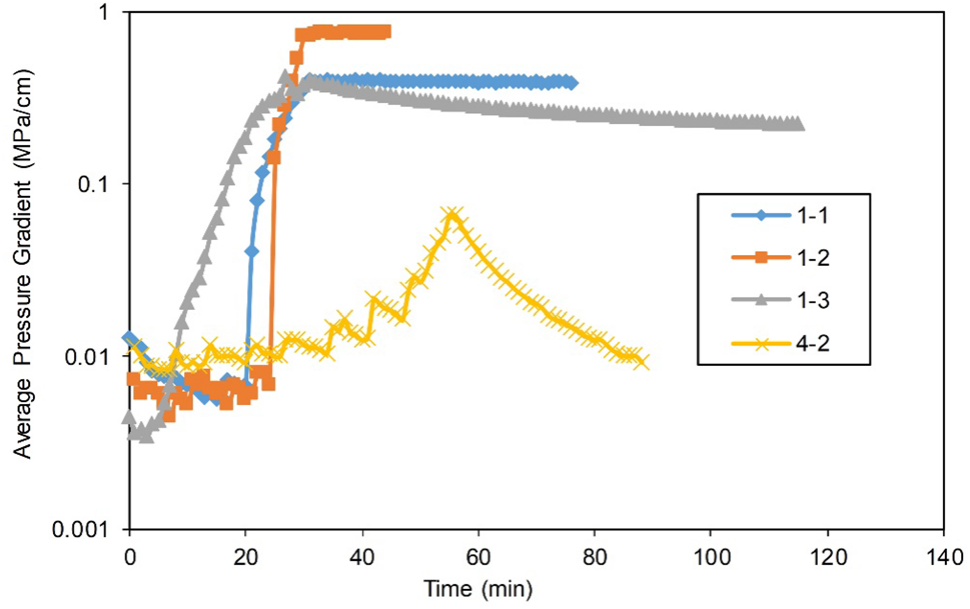
Average pressure gradients at different times in the near-wellbore zone of the Group I.

Figure 7 shows the dynamic pressure drop profile of the volumetric gas reservoir in Experiment 4-2. Figures 8, 9 and 10 show the pressure drop profiles of the water drive in Experiments 1-1, 1-2, and 1-3. For the convenience of analysis, the average pressure gradients in the near-well zones during the above four experiments are calculated and plotted according to the pressure parameters (Experiments 1-1, 1-2 and 4-2 at both ends of Core 4, and Gas Reservoir 1-3 at both ends of the gas reservoir), as shown in Fig. 11.

The experimental results show that the dynamic pressure drop profiles of the volumetric gas reservoir and the WDGRs are significantly different, providing rich information about the gas reservoir dynamics.

The pressures at various positions evenly decreased during the production of the volumetric gas reservoir in Experiment 4-2. When the period of stabilized production ended, the *R* in the reserves was nearly uniform. When the production was stopped at 88 min, the residual pressure at the various positions was nearly completely dissipated, which corresponded to a gas *R* greater than 98%.

Matrix 2 of Gas Reservoir 1-1 had the largest range, and Well 1 was 50 cm from the FZ. The dynamic pressure drop process of the gas reservoir shows that the pressure drop in the entire gas reservoir was relatively synchronous and occurred within the first 20 min of gas production, indicating that the initial reserve development was balanced and that the reservoir could provide a stable gas supply to the gas well. In this stage, the maximum pressure gradient of the gas supply path of the gas reservoir, which occurs in the near-well area of the low-pressure MZ 2 (Core 4), was only 0.007 MPa/cm (Fig. 11). Thereafter, the pressure gradient in the immediate vicinity of the well began to increase rapidly, reaching 0.18 MPa/cm at 25 min and a peak value of 0.34 MPa/cm at 30 min before stable production ended. The significant pressure drop funnel that formed around the gas well indicated that a large amount of formation energy was lost in the immediate vicinity of the well, i.e., the MZ. When Well 1 was abandoned and production was stopped, the pressure gradient near the well finally stabilized at 0.38 MPa/cm, and the residual pressure in the peripheral FZs and MZ 1 (Cores 1-3) was as high as 19.6 MPa to 21.6 MPa. A large amount of residual reserves was not developed due to water sealing. The ${\text{R}}$ of the corresponding gas reservoir was only 37.9%.

Well 1 in Experiment 1-2 was 25 cm from the FZs, 50% of the distance in Experiment 1-1. Similar to Experiment 1-1, the initial pressure drop in Experiment 1-2 was synchronous in the entire gas reservoir. In the later stage, due to the intrusion of formation water in the near-well zone (MZ 2), the pressure gradient of the near-well zone increased rapidly. In the process of production decline, the average pressure gradient of the near-well zone was stabilized at approximately 0.74 MPa/cm, which was the highest value among the three WDGRs, approximately twice that in Experiment 1-1.

Well 1 in Experiment 1-3 was directly connected to the FZs. Its pressure drop profile was significantly different from that of Experiments 1-1 and 1-2. Whether in the early or late stages of development, the *R* in all parts of the gas reservoir Experiment 1-3 was relatively balanced, and the near-well and peripheral pressures gently and synchronously reduced. Due to the high conductivity of the fractures, gas and water were produced together from the gas well, and the energy of the aquifer was simultaneously reduced. This reduces the physical damage due to water invasion, allowing the gas to flow to the gas well through the fractures.

The gas reservoir pressure drop profiles in Figs. 8, 9 and 10 also show that, with only Well 1 in production, the ${\text{R}}$ of the low-permeability MZ 1 (Core 2) surrounded by the FZs at the distal end of the gas well depended on the peripheral FZs (Cores 1 and 3). If the ${\text{R}}$ of the reservoir in the FZ was low (Experiments 1-1 and 1-2), the reserves in MZ 1 were sealed off and could not be developed; if the ${\text{R}}$ of the reservoir in the FZs was high (Experiment 1-3), the MZ 1 ${\text{R}}$ would supply gas to the gas well through the FZs and achieve a high gas reservoir ${\text{ R}}$.

#### Distribution of residual water and reserves

The cores representing the three types of WDGRs were removed immediately after the gas production experiments ended. Then, the average water saturation of the cores in the different parts of the gas reservoirs was obtained using the weighing method, as shown in Table 4.

**Table 4:** Water saturation increment in different zones at the end of Group I (%).

Experimental No	SFZ 1—Core 1	MZ 1—Core 2	LFZ 2—Core 3	MZ 2—Core 4	Average in the gas reservoir
1-1	46.34	27.60	47.47	44.43	41.46
1-2	53.11	21.45	50.73	45.65	42.74
1-3	45.86	9.56	41.56	n/a	32.33

The experiments show that in Experiments 1-1 and 1-2, the overall average water saturations after production were not very different, both exceeding than 40%. Experiment 1-3 with the gas well connected to the FZs produced gas with water for a relatively long time after water breakthrough was observed in the gas well, and the drainage effect was good. The water saturation was only 32.33%, which was approximately 10% lower than that in Experiments 1-1 and 1-2.

All the experimental results obtained for the three types of WDGRs show that the water invasion in the FZs directly connected to the aquifer was the most serious, and correspondingly, the water saturation was the highest, reaching 40–55%. Although near-well MZ 2 was the farthest from the aquifer, its water saturation was also approximately 45%. The high water saturation of MZ 2 indicated that the fracture was the main water intrusion channel and that the water spread into the MZ far from the aquifer via the fracture. This is consistent with the conclusion of Sait from a study of the water invasion mechanism of a fractured carbonate gas reservoir^36^.

MZ 1, surrounded by the FZs, had the lowest average water saturation for each experimental scheme, i.e., 27.60%, 21.45%, and 9.56% in Experiments 1-1, 1-2 and 1-3, respectively, which were considerably lower than those in other zones. The reason for this is that the main water invasion mechanism of MZ 1, the imbibition effect, was different from those of the other zones. Specifically, the permeability of MZ 1 was 0.67 mD, which was considerably lower than that of the reservoirs in the peripheral FZs, where the gas supply rate was slow and the development of the remaining reserves was delayed. The pressure was always slightly higher than that of the connected FZs (this can be verified with the measured pressure data). Physically, gas and water in porous media cannot flow from a low-pressure FZs to a high-pressure MZ. Therefore, the mechanism of the water invasion in MZ 1 can mainly be the imbibition caused by the capillary force.

Gas reservoirs with different geological conditions and even different zones of the same gas reservoirs may have completely different water invasion mechanisms. Their water saturation and residual gas distributions also may be significantly different.

The relative residual reserves of different zones (the ratio of the residual reserves to the total gas reserves) can be calculated after converting the water saturation to the residual gas saturation while considering the residual pressure and porosity of various zones in the gas reservoir. The specific calculation method is as follows:

1 $${\text{f}} = \frac{{{\text{P}}_{{{\text{rk}}}} {\text{S}}_{{{\text{gk}}}} \emptyset_{{\text{k}}} }}{{\mathop \sum \nolimits_{{\text{k = 1}}}^{{4}} {\text{P}}_{{{\text{rk}}}} {\text{S}}_{{{\text{gk}}}} \emptyset_{{\text{k}}} }}.$$

The proportions of residual reserves in different zones after the production of the three types of WDGRs for Group I were calculated and are shown in Table 5.

**Table 5:** Percentage of residual reserves at the end of Group I (%).

Experimental No	SFZ 1—Core 1	MZ 1—Core 2	LFZ 2—Core 3	MZ 2—Core 4
1-1	27.93	32.08	28.51	11.48
1-2	26.93	38.05	29.56	5.46
1-3	29.48	39.69	30.83	n/a

Table 5 shows that the distribution of the residual reserves in different zones of the same gas reservoir was not uniform. Although the porosity of MZ 1 was not high in the three experiments, the residual reserves of this zone were apparently higher than those of the other zones, taking a high saturation of residual gas into account. The amount of residual reserves in the near-well MZ 2 in the near-well area of Experiments 1-1 and 1-2 was the lowest. The reserves were concentrated in the peripheral FZs and MZ 1, and the gas reservoir reserves were not recovered uniformly; the difference among the proportions of the residual reserves in the different zones of Experiment 1-3 is no more than 10%, and the reserve development was the most balanced.

### Impact of drainage positions

The multiwell drainage gas production process is often adopted in the early stage of development in fractured WDGRs. For fractured reservoirs or high-permeability zones connecting to the aquifer, water drainage wells (such as Well Chi 27 in Fig. 1 and Well 2 in Fig. 2) are deployed to block aquifer water intrusion. In Group II, the impact of the drainage position on the water control effect was simulated by adjusting the position of the gas production well. As shown in Table 2 and Fig. 3, Well 2 in Experiments 2-1 and 2-2 was set at Point A in the middle of the LFZ and Point B near the water edge, respectively (the distance between Points A and B was 12.5 cm). Well 2 in Experiments 2-3 and 2-4 was located in the zone with small fractures, at Point C and Point D. In the experiments, the operation of the Well 1 and Well 2 was simultaneously initiated. For joint gas production and water draining, the well that reached an abandoned production rate first, had to keep open until the other well reached the abandonment production rate.

#### Production performance

**Figure 12:**
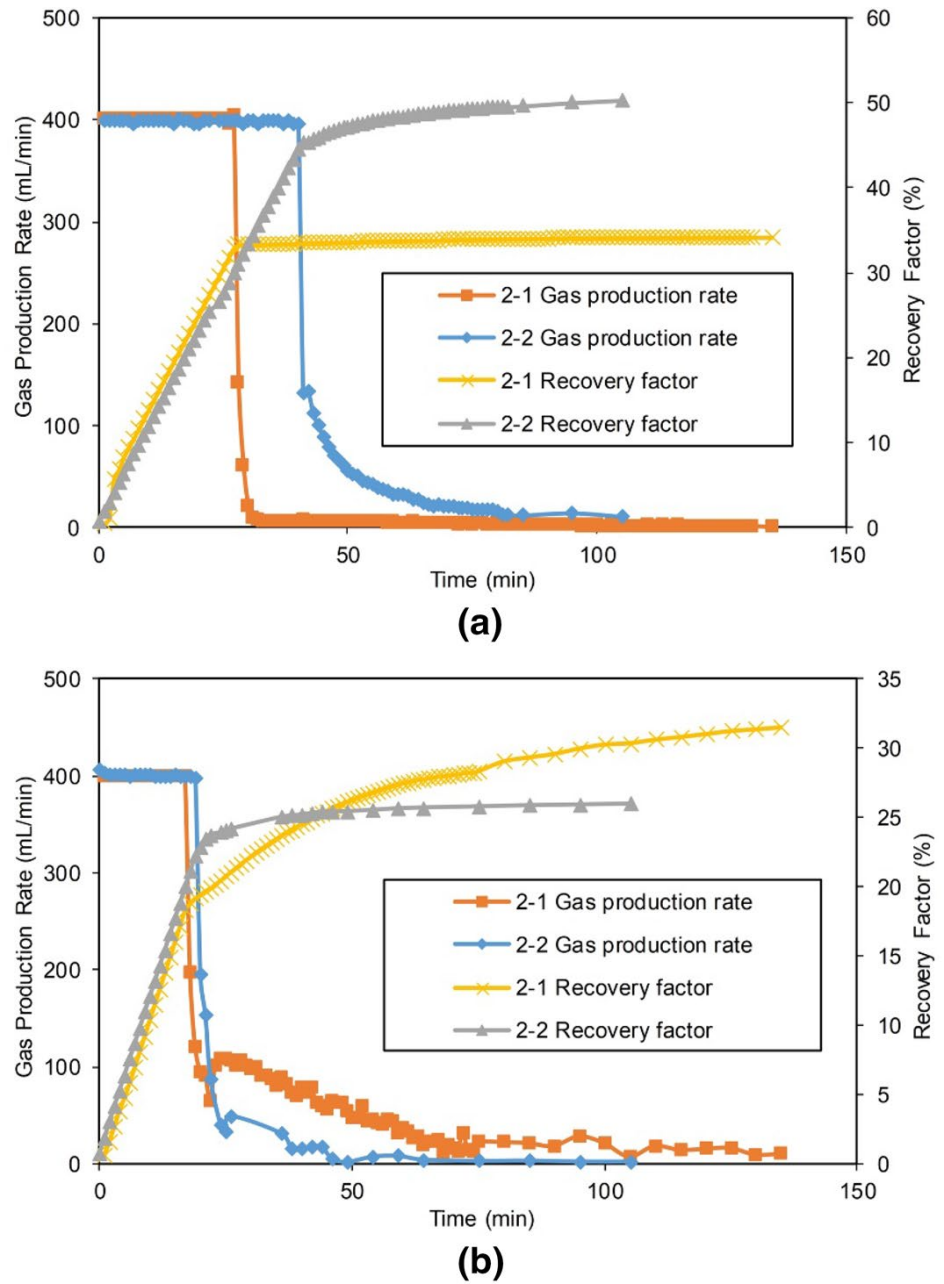
Gas production of two wells in Experiments 2-1 and 2-2: (**a**) gas production of Well 1 located in the MZ, and (**b**) gas production of Well 2 located in the FZ.

**Figure 13:**
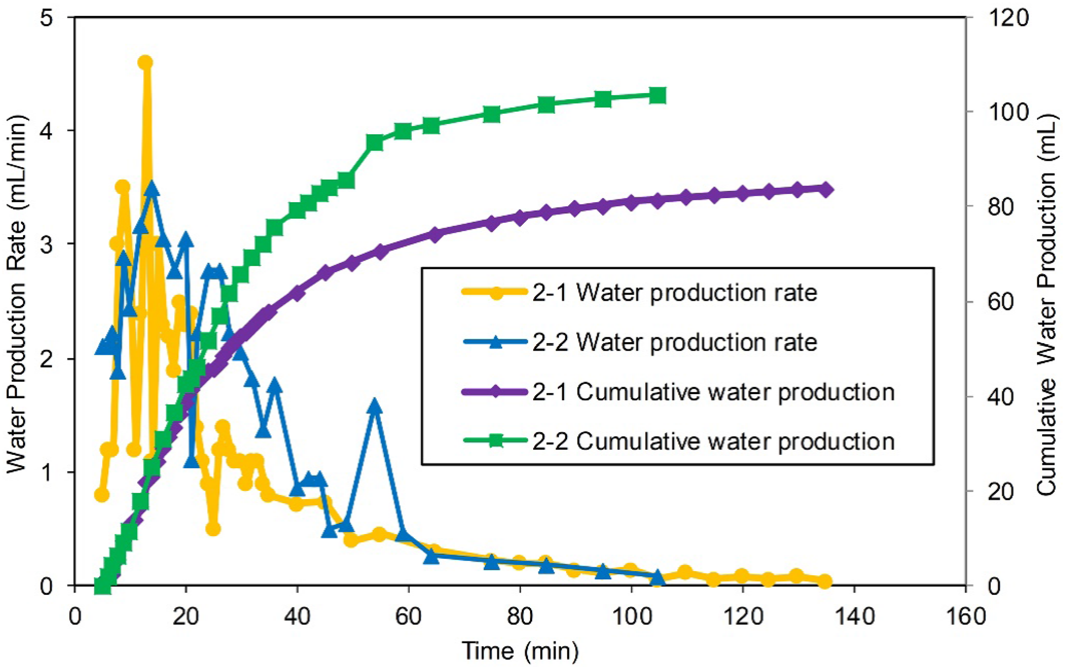
Water productions of Well 2 in Experiments 2-1 and 2-2.

Experiments 2-1 and 2-2 in which the drainage well was set in a LFZ were selected for a comparative analysis. The gas production and water production results of both experiments are shown in Figs. 12 and 13. The production parameters are shown in Table 6.

**Table 6:** Statistics on the production of Group II.

Experimental No	Gas Well 1 in the distal MZ			Gas Well 2 in the FZ				Whole gas reservoir		
	Stable production period, min	Abandonment time, min	Single well ${\text{R}}$, %	Breakthrough time, min	Stable production period, min	Abandonment time, min	Single well ${\text{R}}$, %	Production period, min	Cumulative water production, mL	${\text{R}}$, %
2-1	27	29	37.46	5	17	135	35.44	135	83.9	72.79
2-2	40	105	50.29	5	19	44	28.58	105	103.8	78.87
2-3	25	34	33.53	11	23	144	44.44	144	80.2	78.08
2-4	26	65	35.44	6	14	175	44.78	175	86.9	80.33

Since multiwell water control was conducted in the early stage, the gas ${\text{R}}$ of Experiments 2-1 and 2-2 was significantly improved compared with that of Experiment 1-1 with a single production well, Well 1, reaching 72.79% and 78.87%, respectively (the gas reservoir conditions were exactly the same, and the ${\text{R}}$ of Experiment 1-1 was only 37.9%). This shows that in the early stage of development, the gas wells in reasonable positions perform joint drainage, greatly reduce the impact of the water invasion and significantly improve the gas recovery.

The production curve shows that no water breakthrough occurred during the production of Well 1 in the distal MZ in Experiments 2-1 and 2-2 due to the high-efficiency drainage of Well 2. This indicates that joint drainage and gas production effectively prevented the aquifer from invading into the deep part of the gas reservoir. In Experiment 2-2, Well 2 was closer to the aquifer and had a higher drainage capacity and a lower gas production capacity. The ${\text{R}}$ of Well 2 was 28.58%, which was only 80.6% of that in Experiment 2-1. Experiment 2-2 had a better effect on the overall water control of the gas reservoir. The stable production period of Well 1 at the distal matrix position reached 40 min, and the ${\text{R}}$ was as high as 50.29%, which were 1.48 times and 1.34 times those of the corresponding Well 1 in Experiment 2-1, respectively. The ${\text{R}}$ of the entire gas reservoir reached 78.87%, which was 6.08% higher than that in Experiment 2-1.

Clearly, the drainage gas recovery in Experiments 2-1 and 2-2 was better than Experiment 1-1, not considering the technical limitations and economic costs.

Both production parameters of Experiments 2-3 and 2-4 are tabulated in Table 6. The ${\text{ R}}$ of the gas reservoirs reached 78.08% and 80.33%, respectively, which were also greatly improved compared with the single-well production in Experiment 1-1: the differences in the production performance metrics of Experiment 1-1 and Experiments 2-1 and 2-2 were noticeable. The impact of the drainage position in the SFZ was small, and the difference in the ${\text{R}}$ did not exceed 2.5%. Water-control Experiments 2-3 and 2-4 had similar drainage effectiveness based on the drainage and gas production capacities of the main drainage well, Well 2. Thus, their drainage functions and the protective effects on the gas reservoirs were not considerably different. Compared with drainage Well 2, which was set in the LFZ (Experiments 2-1 and 2-2), Well 2 in the SFZ (Experiments 2-2 and 2-3) had a delayed breakthrough time, and its drainage speed was slower, but the corresponding gas production capacity increased, and the ${\text{R}}$ reached 44%, which was more than 10–15% higher than that of Experiments 2-1 and 2-2.

Group II showed that the gas drainage effectiveness and the gas production capacity were significantly affected by the location of the gas well upstream, performing the main drainage function. The distance from the gas well to the aquifer and the seepage capacity of the reservoir in the area were important factors. At the same time, these results also illustrated the complexity of the development of a water control plan. The drainage position should be determined by comprehensively considering the recovery of the entire gas reservoir, the technical conditions on site and the economic benefits.

#### Water invasion analysis

**Figure 14:**
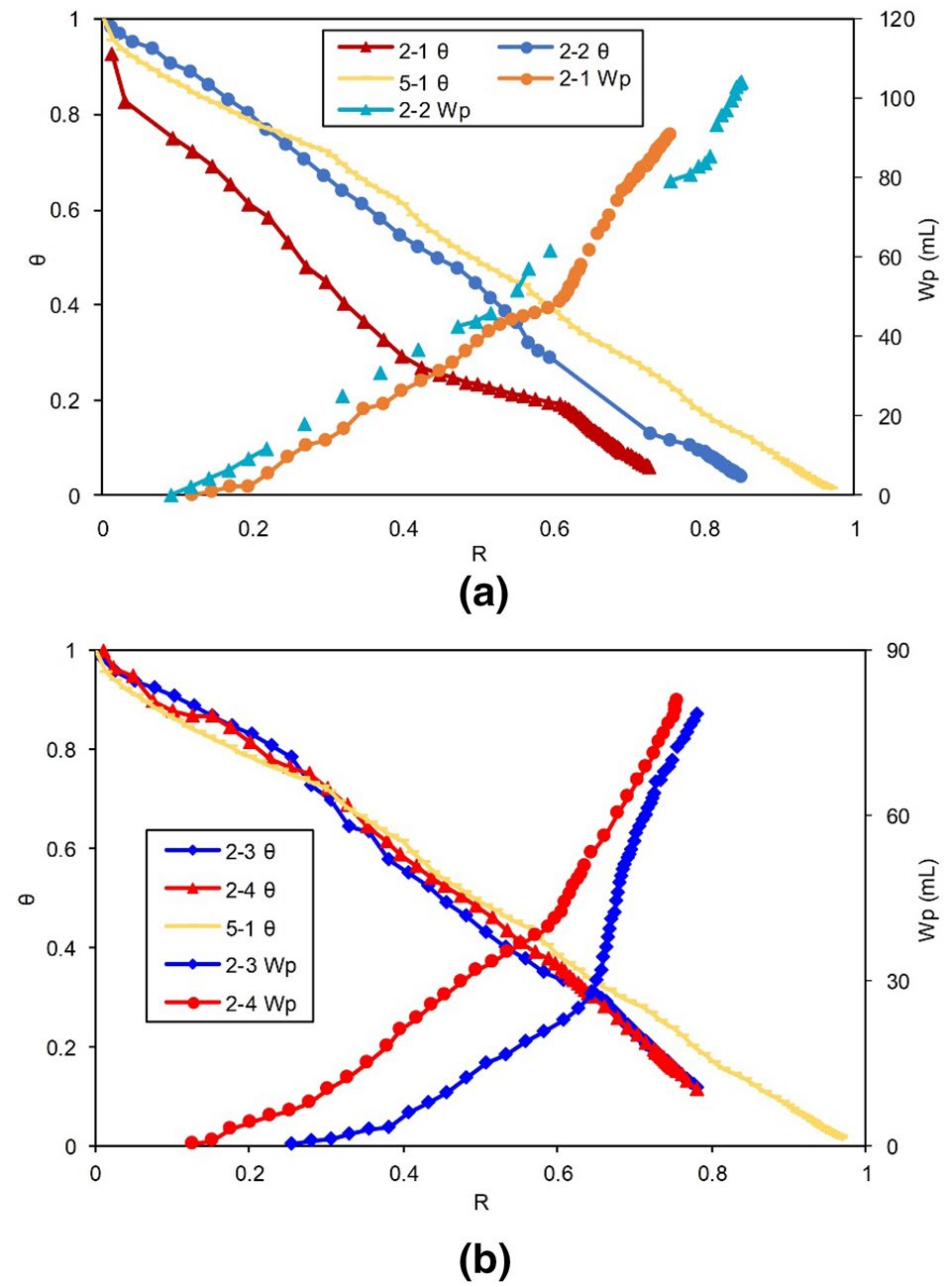
Relationship between relative apparent pressure of the formation and ${\text{R}}$: (**a**) drainage Experiments 2-1 and 2-2 in the LFZ, and (**b**) drainage Experiments 2-3 and 2-4 in the SFZ.

The $\uptheta \sim {\text{R }}$ curves of Group II are included in Fig. 14. For comparison, the $\uptheta \sim {\text{R }}$ curves of the volumetric gas reservoirs using the same model (basic Experiment 5-1) are also shown in Fig. 14. For the convenience of analysis, the relationships between the ${\text{W}}_{{\text{p}}}$ and the ${\text{ R}}$ of the reserves for the corresponding experiments are also plotted in Fig. 14.

The $\uptheta \sim {\text{R }}$ curves of Group II showed the characteristics of water drainage. As Well 2 in Experiments 2-1 and 2-2 produced water earlier and faster, the relative pressure curve started to decrease earlier, and the rate of decrease was greater, implying a stronger drainage effect. As the production progressed, the curves decreased to greater extents, indicating that as the drainage proceeded, the net water influx was reduced and converted to net water production in the later stage.

In Fig. 14, the upward ${\text{W}}_{{\text{p}}} \sim {\text{R}}$ curve indicates that the unit gas production corresponded to an increase in both the water production and the water–gas ratio. The water production of the four experiments significantly increased once the ${\text{R}}$ was greater than 50–60%. Thus, the water–gas ratio of the gas reservoir in the later stage of production increased significantly, which also indicates that only the large-scale drainage in the later stage could maintain the gas production of the gas well. Meanwhile, the corresponding relationship between the ${\text{W}}_{{\text{p}}}$ and the relative formation pressure decline curve was obvious, indicating that the $\uptheta \sim {\text{R }}$ relation curve could accurately reflect the water invasion degree of the gas reservoir in a timely manner.

#### Dynamic pressure drop

**Figure 15:**
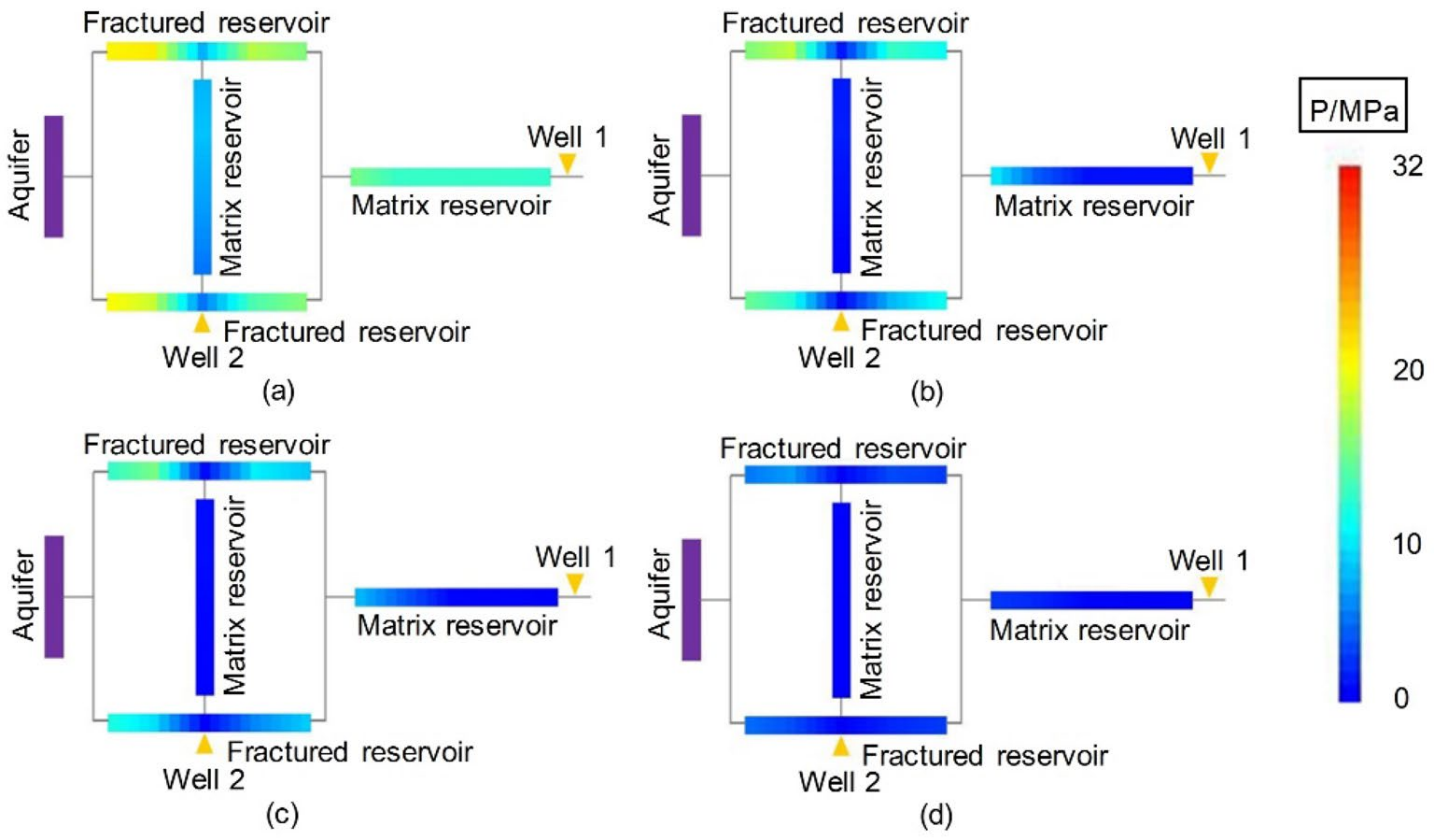
Pressure distributions at different stages of Experiment 2-1 (Well 2 is at Point A): (**a**) produced for 10 min, (**b**) produced for 20 min, (**c**) produced for 30 min, and (**d**) gas reservoir production halted at 135 min.

**Figure 16:**
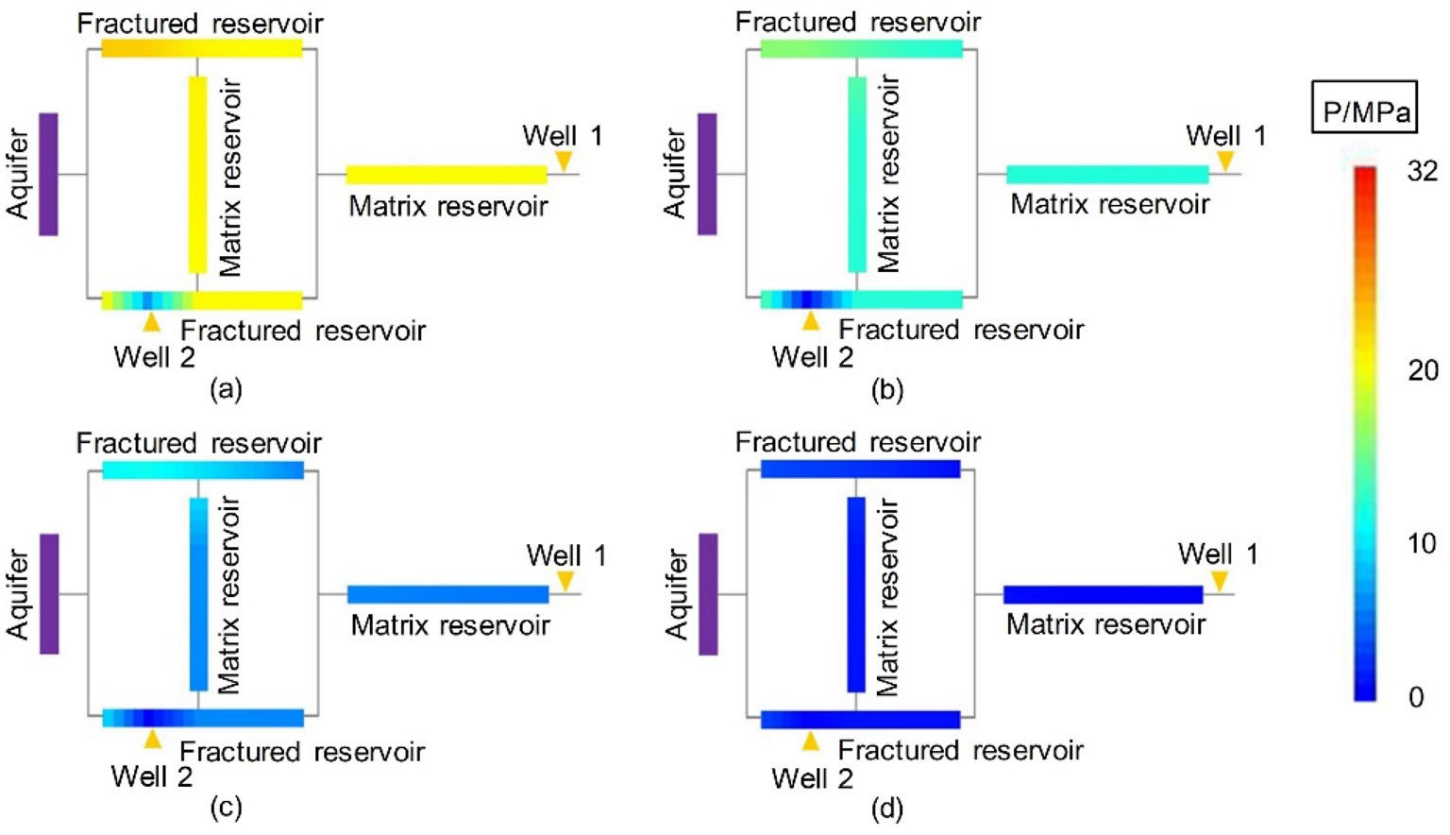
Pressure distributions at different stages of Experiment 2-2 (Well 2 is at Point B): (**a**) produced for 10 min, (**b**) produced for 20 min, (**c**) produced for 30 min, and (**d**) gas reservoir production halted at 105 min.

The dynamic pressure drop profiles of Experiments 2-1 and 2-2 are included in Figs. 15 and 16. Since water control was performed via both wells in an early stage of gas reservoir development, the pressure profiles of the gas reservoirs of Experiments 2-1 and 2-2 decreased rapidly and simultaneously. The residual pressure was low, which was significantly different from that of Experiment 1-1, as plotted in Fig. 8. The high pressure drop funnel that formed in the immediate vicinity of Well 1 in Experiment 1-1 was not observed in Experiments 2-1 and 2-2. This shows that for a fractured WDGR, multiwell water control conducted in the early stage could effectively prevent the aquifer water from intruding the gas reservoir, protect the entire gas reservoir, and greatly improve the ${\text{R}}$ and balance of the gas reservoir development.

As Well 2 was deployed at a different position, the order of development of the remaining reserves in different zones of the gas reservoir changed. In Experiment 2-1, Well 2 was at Point A, in the middle of the LFZ connected to MZ 1. MZ 1 supplied the most gas to Well 2 through the fracture, and the average pressure in that zone dropped rapidly, close to the rate of pressure drop in MZ 2 connected to Well 1. After producing for 20 min, the residual pressure almost reached vanished. In Experiment 2-2, Well 2 was closer to the aquifer, and its drainage capacity was higher, so the pressure in the other areas of the gas reservoir, including the SFZ and the MZ in the upper part of the gas reservoir, was simultaneously reduced, which protected the entire gas reservoir more effectively.

#### Distribution of residual water and reserves

Table 7 provides the average water saturation of the gas reservoir in Group II, which was 22.4% when production ended and significantly lower than that in Experiment 1-1 (41.46% water). Therefore, in the early stage, performing multiwell joint drainage and gas production can effectively prevent water invasion.

**Table 7:** Water saturation increment in different zones at the end of Group II (%).

Experimental No	SFZ 1—Core 1	MZ 1—Core 2	LFZ 2—Core 3	MZ 2—Core 4	Average water content
2-1	31.99	4.46	31.32	23.46	22.81
2-2	33.01	6.78	23.78	1.91	16.37
2-3	38.09	22.91	33.51	14.78	27.32
2-4	23.27	13.49	41.12	14.99	23.22

Comparing the experimental schemes, such as the large-fracture drainage Experiments 2-1 and 2-2, the average water saturation of the gas reservoir after production was 22.81% and 16.37%, which were lower than 27.32% and 23.22% of the small-fracture drainage Experiments 2-3 and 2-4 and indicated that drainage in the large-fracture can more greatly restrain water invasion.

The production parameters in Table 6 show that the drainage well located in a large-fracture (i.e., Experiments 2-1 and 2-2) will produce water earlier and more frequently. Based on the above analysis of Fig. 14, Well 2 in Experiments 2-1 and 2-2 produces water earlier and faster than that in Experiments 2-3 and 2-4, so its *θ* ~ *R* curve dips earlier and its dip amplitude is larger, implying a stronger drainage effect. Both Table 6 and Fig. 14 show that this finding is reliable and accurate, since the drainage effect of large fractures is better and more effective at avoiding water intrusion into the deeper part of the gas reservoir.

When the water is drained at the near-aquifer location in the FZ (Experiments 2-2 and 2-4), the increment of water saturation in the gas reservoir is small, approximately 5–7% lower than that drained in the middle of the FZ (Experiments 2-1 and 2-3, far from the aquifer). This shows that whether the reservoir permeability is high or low, the drainage of water near the aquifer can better prevent water invasion.

Notably, due to the high-efficiency drainage in the early stage of the experiments, all four experiments successfully protected the low-permeability MZ 1 surrounded by fractures and the low-permeability MZ 2 at the toe of the well. In the large-fracture drainage Experiments 2-1 and 2-2, the water saturation of MZ 1 was increased by only approximately 5%, and almost no water invasion occurred. In Experiment 2-2, the water content of the distal MZ 2 was increased by only 1.91%.

In general, although the MZ had poor physical properties and low permeability, its ${\text{R}}$ on the whole was higher than that of the FZ connected to the aquifer. This indicated that, on the one hand, due to multiwell drainage, the MZ was basically unaffected by water invasion; on the other hand, as the number of gas wells increased, the gas supply distance from the MZ to the gas wells was greatly reduced, so the reserves could be developed more easily (Table 8).

**Table 8:** Percentage of residual reserves in different zones at the end of Group II (%).

Experimental No	SFZ 1—Core 1	MZ 1—Core 2	LFZ 2—Core 3	MZ 2—Core 4
2-1	48.62	0.61	36.47	14.30
2-2	42.68	25.76	25.29	6.27
2-3	21.37	28.09	40.49	10.05
2-4	26.13	27.46	35.10	11.30

In Experiments 2-1 and 2-2, which drained water along the large fractures, the ${\text{R}}$ of the reserves in LFZ 2 was greater than that in SFZ 1, which was far from Well 2. However, in Experiments 2-3 and 2-4, which drained water along the small fractures, the ${\text{R}}$ in SFZ 1 was better than that in LFZ 2, which was far from Well 2.

### Impact of drainage timing

As shown in Table 2, Experiments 3-1 and 3-2 were conducted with Geological Model 2. At the beginning of Experiments 3-1 and 3-2, only a single well, Well 1, was put into production. Once Well 1 reached the abandonment production rate, Well 2 was immediately opened in Experiment 3-1. The drainage well was set in the middle of the LFZ, and it was produced simultaneously with Well 1. In Experiment 3-2, Well 1 was first shut in and then opened to produce gas 16 h later. When the production of Well 1 decreased to the abandonment production rate again, drainage Well 2 was set at Point A in the middle of the LFZ and was put on production. The production process lasted until the production of both wells was reduced to the abandonment production rate. Experiment 3-3 was implemented with Geological Model 3. Well 1 was directly connected to the fracture zone (FZ) through the high-conductivity fault zone. Its drainage timing was the same as that of Experiment 3-1.

The above experimental process reflects that the first stage of production in Experiments 3-1 and 3-2 was identical to that in Experiment 1-2 (with the same gas reservoir) for single-well (Well 1) production, except that in the second stage, the increases in Well 2 were added. Similarly, the first stage of Experiment 3-3 was identical to that in Experiment 1-3 of single-well (Well 1) production (for the same gas reservoir). Therefore, the production situation of the first stage of Group III is not repeated.

#### Production performance

**Figure 17:**
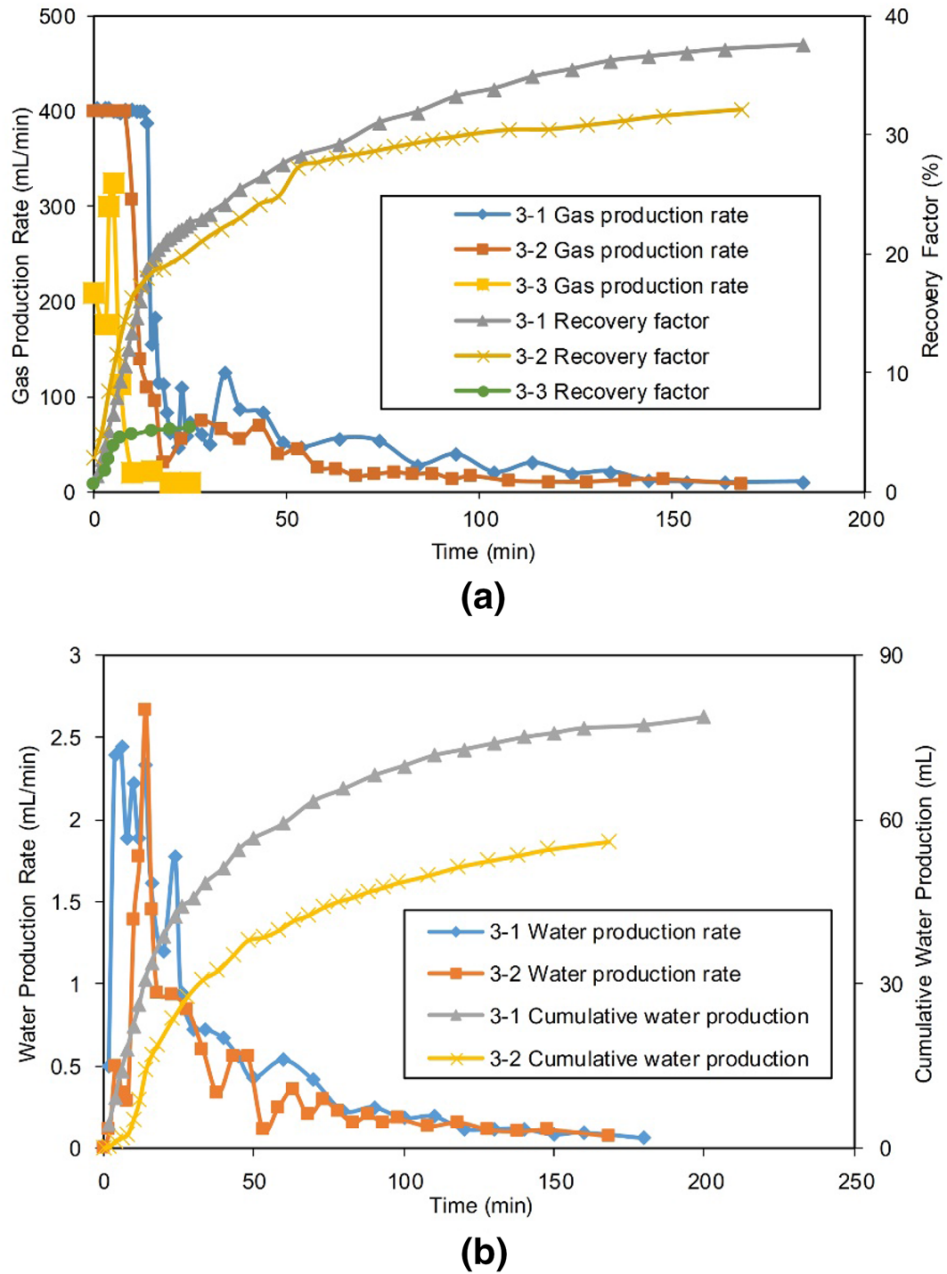
Production of Gas Well 2 in the second stage of Group III: (**a**) gas production of the gas Well 2, and (**b**) water production of the gas Well 2.

The production curve and the production parameters after adding Well 2 in the second stage of production of Group III are shown in Fig. 17 and Table 9, respectively.

**Table 9:** Statistics on the production of Group III.

Experimental No	First stage ${\text{R}}$, %	The Joint production of two wells in the second stage								Cumulative ${\text{R}}$ of the two stages, %
		Gas Well 2 in the FZ				Matrix Well 1				
		Stable production period, min	Abandonment time, min	${\text{R}}$, %	${\text{W}}_{{\text{p}}}$, mL	Stable production period, min	Abandonment time, min	${\text{R}}$, %	${\text{W}}_{{\text{p}}}$, mL	
3-1	41.4	14	184	37.6	78.6	0	30	0.9	1.1	79.9
3-2	41.4	10	168	31.7	56.1	0	20	2.9	3.6	76.0
3-3	62.5	1	25	5.4	0.33	0	12	0.8	2.5	68.7

As shown in Table 9, the gas production and ${\text{W}}_{{\text{p}}}$ in the original production well, the remote Well 1, were extremely small in the second stage of each of the three experiments. By the end of production, the ${\text{R}}$ of Well 1 was less than 3%, and the ${\text{W}}_{{\text{p}}}$ was less than 4 mL, which can be neglected. The main gas production could be attributed to the later addition of Well 2.

In the second stage of Experiment 3-1, the ${\text{R}}$ of Well 2 reached 37.6%. The cumulative ${\text{R}}$ of the two stages was 79.9%.

After the wells were shut in for 16 h in Experiment 3-2, Well 1 was initiated first, in the stage with a ${\text{R}}$ of 2.9%. The water produced was only 3.6 mL. This showed that the water invasion into the gas reservoir formed a strong water seal, making it difficult for gas and water to flow into Well 1. It was difficult to effectively resume the production of Well 1 after a long-term shut in. After that, Well 2 was added, and the ${\text{R}}$ sharply reached 31.7%, which implies effective stimulation. In the two stages, the cumulative ${\text{R}}$ increased to 76%, which was slightly lower than that in Experiment 3-1.

Experiments show that when a single well (Well 1) was put into production, once it was flooded, its production would decrease rapidly and could not be recovered by itself. However, by adding a new gas well, Well 2, in the high-permeability zone of the gas reservoir, the water-sealed reserves in the gas reservoir could be unlocked, which significantly improved the gas ${\text{R}}$ (38.45% and 34.6%). In addition, the later the drainage timing of the drainage well, the more serious the water invasion, the more difficult the development of the water-sealed reserves, and the lower the ${\text{R}}$ of the gas reservoirs.

Compared with the outstanding stimulation effect of adding Well 2 in Experiments 3-1 and 3-2, the gas and ${\text{Q}}_{{\text{w}}}$ in the second stage of Experiment 3-3 were extremely low. The ${\text{R}}$ was only 5.4%, and the cumulative ${\text{R}}$ of the two stages was 68.7%, which was far lower than that achieved in Experiments 3-1 and 3-2. Well 1 in the gas reservoir of Experiment 3-3 was directly connected to the FZ via a high-conductivity fault zone, where the energy in the gas reservoir was largely consumed in the first stage (the residual relative apparent pressure in Experiment 1-3 was only 0.25, in Fig. 6), which was not enough to produce gas from the water-sealed zone; thus, it is not possible to significantly increase the gas production in such gas reservoirs by adding gas wells.

#### Dynamic pressure drop

**Figure 18:**
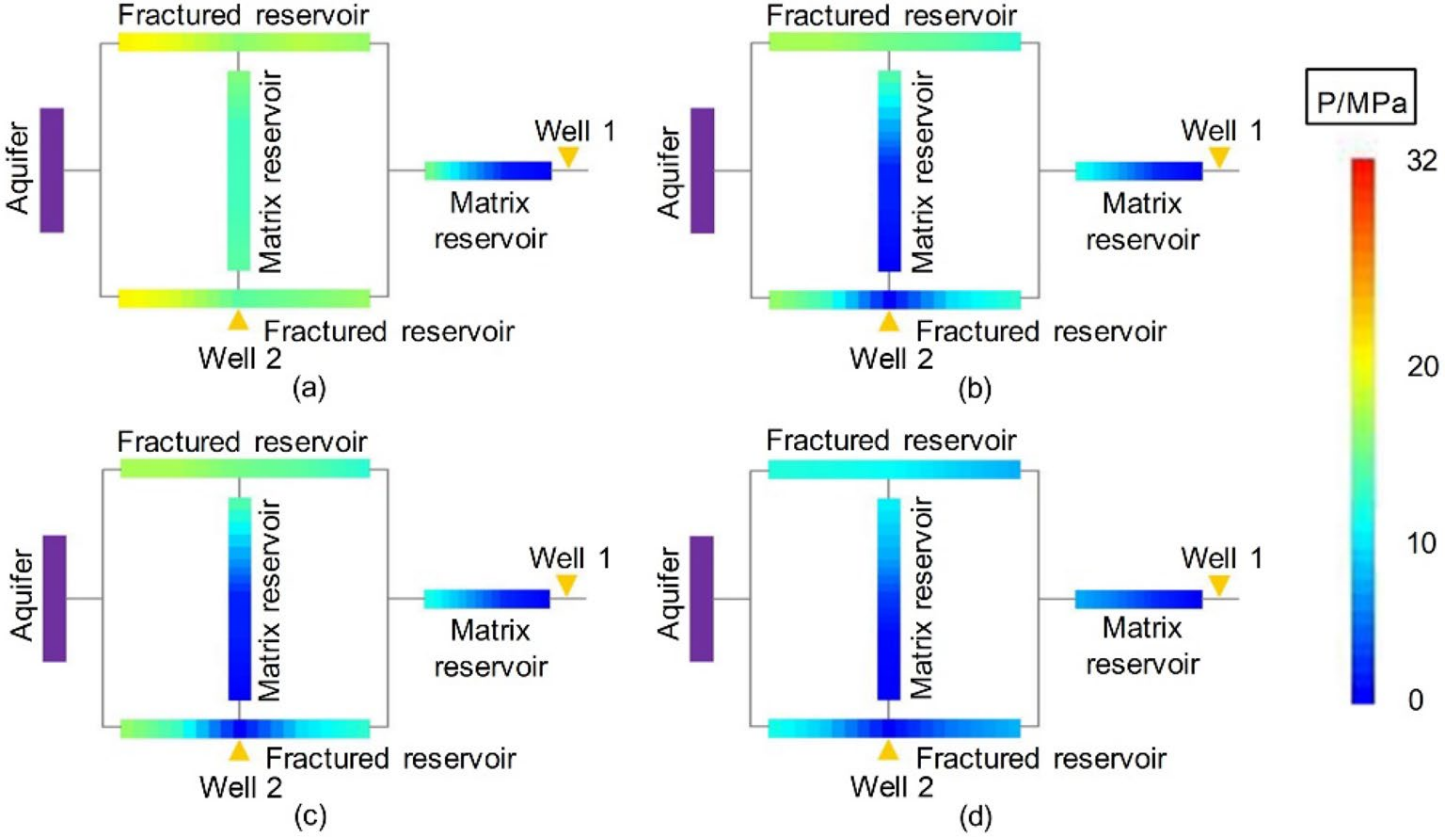
Pressure distributions at different times in the second stage of Experiment 3-1 (after drainage): (**a**) Well 2 started up—5 min, (**b**) end of the stabilized period of Well 2–14 min, (**c**) late production period—40 min, and (**d**) gas well production halted—184 min.

The dynamic pressure drop profiles of the second stage of Experiment 3-1 are shown in Fig. 18 (for the first stage, see Experiment 1-2, shown in Fig. 9).

In the second stage of Experiment 3-1, after adding Well 2 and producing from the two wells for 5 min, the pressure drop profile changed. When Well 2 was put into production, the residual reserves in MZ 1 were quickly produced. Since the gas production at Well 2 was stable, a large amount of gas and water was produced. Specifically, the peripheral areas that were difficult to exploit from Well 1 in the first stage were developed by Well 2 (Cores 1-3). Well 2 continued to produce for 184 min, and the pressure drop profile at the end of the experiment (Fig. 18d) showed that the gas reservoir was relatively balanced and effectively developed.

At the beginning of the second stage of Experiment 3-2, Well 1 was produced first, and the yield stimulation effect was poor, so the pressure drop profile did not change considerably. After adding drainage Well 2, the change in the pressure drop profile was almost the same as that of Experiment 3-1. To avoid repetition, those results are not described in this paper.

Since the drainage scheme in the second stage of Experiment 3-3 almost failed, the relative formation pressure was unchanged from that of Experiment 1-3, and the pressure profile was also stabilized after the first stage of production.

#### Distribution of residual water and reserves

The water saturations for different zones after gas reservoir production are presented in Table 10. Compared with the four experiments in Group II, in which two wells were combined to control water in the early stage of the experiments, Group III showed that the average water saturation increased to more than 30% (from the 22.4% observed for Group II) due to the delay in the drainage well initiation. This indicates that a delayed drainage led to an increase in the net water influx in the gas reservoir.

**Table 10:** Water saturation increment in different zones at the end of Group III (%).

Experimental No	SFZ 1—Core 1	MZ 1—Core 2	LFZ 2—Core 3	MZ 2—Core 4	Average in the gas reservoir
3-1	37.42	19.74	38.86	28.87	31.22
3-2	40.11	17.74	36.02	41.20	33.77
3-3	45.33	8.05	38.99	n/a	30.79

In Experiments 3-1 and 3-2, after the second stage of production, the average water content of the gas reservoir decreased to 31.22% and 33.77%, respectively, which were 11.52% and 8.97% lower than that of the first stage (Experiment 1-2, 42.74%), respectively. Thus, by increasing the effective drainage of the drainage well, the water saturation of the reservoir was reduced and the flow resistance was reduced, which laid the foundation for the residual reserves to be developed.

Compared with Experiment 3-1, in Experiment 3-2, Well 1 was shut in, the drainage was delayed, and the average water saturation of the gas reservoir was increased by 2.5%. The maximum increase was in MZ 2 (where Well 1 located), indicating that during the shut in of Well 1, the gas and water were rebalanced under the enormous pressure difference in the near-well area and continued to flow to the near-well area.

In Experiment 3-3, at the end of the first stage (Experiment 1-3) of production, the average water content was 32.33%. In the second stage, the water drainage had little effect, as only a small amount of gas and water was produced. The average water saturation dropped by only 1.54%.

Compared with Experiment 3-1, the residual reserves of MZ 2 in the near-well area of Well 1 in Experiment 3-2 were increased due to the late timing of the drainage, and the gas and water in the gas reservoir were rebalanced within 160 min after Well 1 was shut in. Under the condition of an enormous pressure difference in the near-well area, the gas and water continuously flowed into MZ 2, which caused the gas and water contents to greatly increase, and the average residual pressure increased by 4.0 MPa. The increase in the water saturation led to an increase in the gas and water seepage resistance, and the difficulty of the development of the remaining reserves increased. Therefore, the proportion of residual reserves in MZ 2 in Experiment 3-2 was significantly greater than that in Experiment 3-1 (Table 11).

**Table 11:** Distribution of residual reserves in different zones at the end of Group III (%).

Experimental No	SFZ 1—Core 1	MZ 1—Core 2	LFZ 2—Core 3	MZ 2—Core 4
3-1	41.07	23.08	26.95	8.90
3-2	40.03	14.58	27.93	17.45
3-3	35.64	34.56	29.80	n/a

In Experiment 3-3, since the second stage of water drainage had little effect, there is no significant difference in the distribution of residual reserves between the second stage and the first stage (Experiment 1-3).

### Impact of the aquifer size

As shown in Table 2, Experiments 4-1 and 4-2 and Experiment 3-1 were conducted using Geological Model 2 under conditions of an infinite aquifer, no aquifer, and an aquifer 15 times greater than the reservoir. In the early stage of Experiments 4-1 and 3-1, only distal Well 1 was produced. After production in Well 1 was reduced to the abandonment production rate, Well 2, in the middle of the LFZ, was initiated, and the production was combined with Well 1. Once both wells reached the abandonment production rate, the production was stopped. Since Experiment 4-2 considered a volumetric gas reservoir, it was developed only by Well 1.

#### Production performance

**Figure 19:**
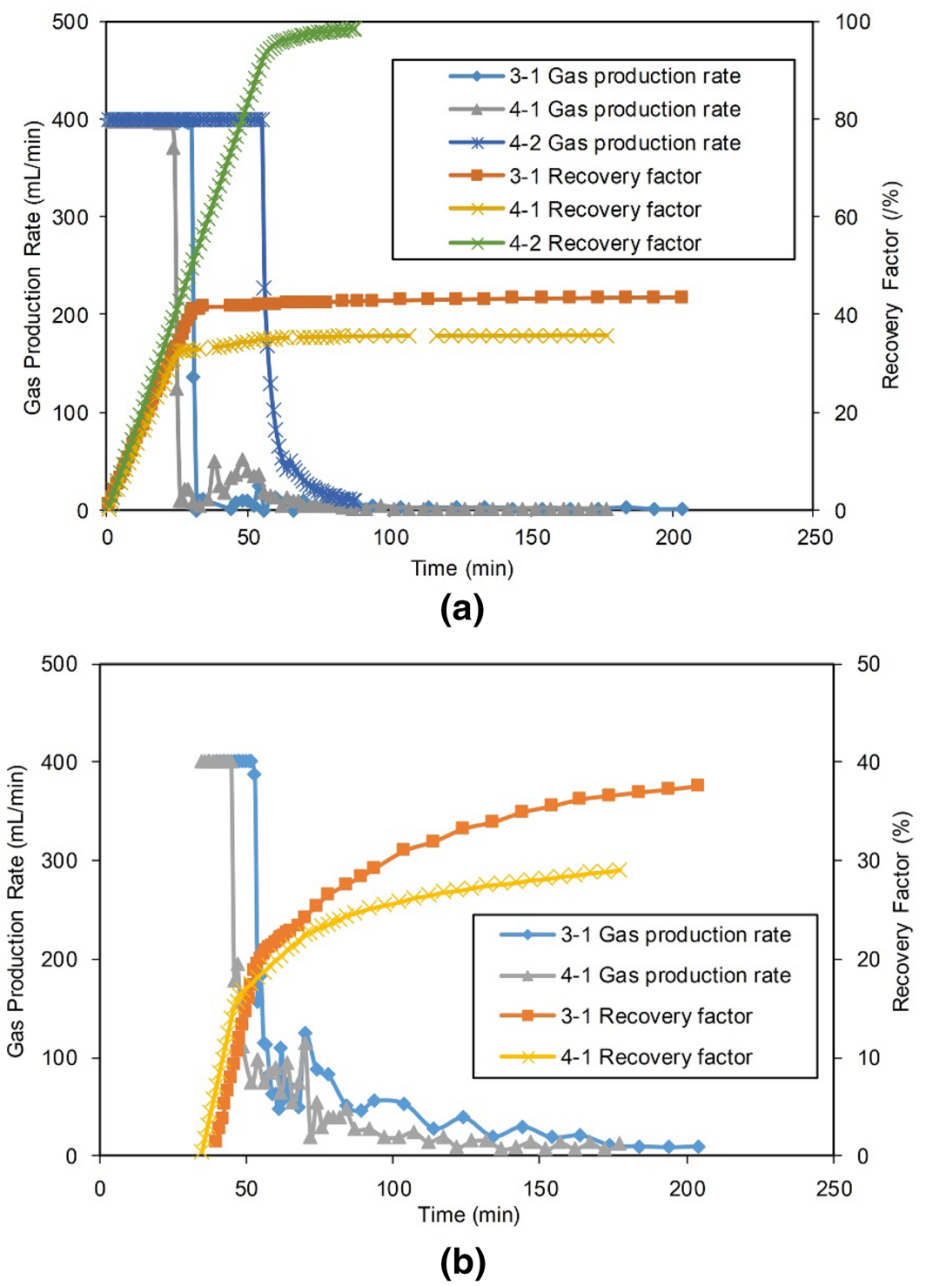
Gas production performance of Experiments 3-1 and 4-1: (**a**) Gas production of Gas Well 1 in the MZ, and (**b**) Gas production of the Gas Well 2 at point A.

**Figure 20:**
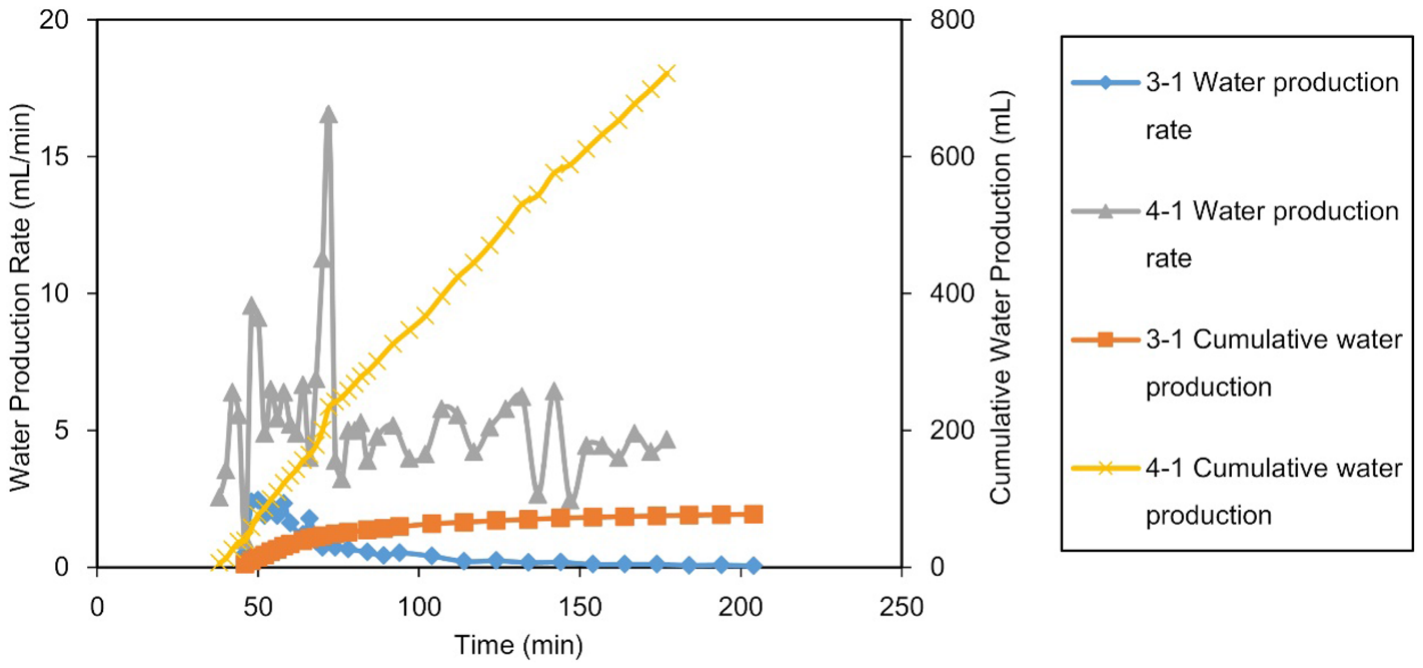
Water production of Gas Well 2 in the FZ of Experiments 3-1 and 4-1.

The production of Experiments 3-1, 4-1 and 4-2 are shown in Figs. 19 and 20, and the production parameters are shown in Table 12.

**Table 12:** Statistics on the production of Group IV and Experiment 3-1.

Experimental No	Gas Well 2 in the FZ				Gas Well 1 in the distal MZ					Cumulative ${\text{R}}$ of the reservoir, %
	Stable production period, min	Downtime, min	Single well ${\text{R}}$, %	${\text{W}}_{{\text{p}}}$, mL	Stable production period, min	First abandonment time, min	Downtime, min	Single well ${\text{R}}$, %	${\text{W}}_{{\text{p}}}$,mL	
3-1	14	184	37.6	78.6	30	49	184	42.9	1.1	80.45
4-1	10	142	29.1	721.7	23	35	142	35.4	14.4	64.50
4-2	Dry well				55	88	88	98.6	0	98.6

Comparing the gas production performance, in the first stage, Well 1 in the MZ in Experiment 3-1 (finite aquifer) exhibited stable production for only 30 min. After 49 min of production, the abandonment production rate was reached, and the ${\text{R}}$ of the single well was 41.4% (42.9% in total, combined with the second stage). However, for the infinite aquifer in Experiment 4-1, due to the high energy of the aquifer, the water invasion was faster and more serious, which led to a shortened (only 23 min) stable production period of Well 1 in the MZ. After 35 min of production, the abandonment production rate was met, and the single-well ${\text{R}}$ was only 33.4% (35.4% in total combined with the second stage).

In the second stage, the same drainage measures were utilized for the same drainage timing used for Experiments 3-1 and 4-1. In Experiment 3-1, the finite aquifer drained from Well 2 with a stable production for 14 min with a single-well ${\text{R}}$ of 37.6%. In Experiment 4-1, the water drainage was fully developed in Well 2 for the infinite aquifer, and the average drainage rate was 12 times larger than that of the finite aquifer (according to Table 12), so only 10 min of stable production were observed. The single-well ${\text{R}}$ was only 29.1%, which was 8.5% lower than that of Well 2 for Experiment 3-1.

At the end of production, the cumulative gas recovery of the infinite aquifer case was 64.5%, which was 15.95% less than that of the finite aquifer case. The single-well stable production period of the volumetric gas reservoir in Experiment 4-2 was as long as 55 min, which was more than twice that in Experiment 3-1, and the ${\text{R}}$ was over 98%.

The above experiments show that the aquifer size had a significant impact on gas recovery. For the same geological and production conditions, the larger the aquifer was, the lower the ${\text{R}}$. Fang et al. performed water invasion experiments with small fractured cores at different aquifer scales. Their research conclusion is consistent with that from this work, which shows that this conclusion may be universal for fractured gas reservoirs^21^.

Comparing the water production performance, in Experiments 3-1 and 4-1, Well 2 was located in the LFZ with high conductivity and directly connected with the aquifer. Therefore, after Well 2 was initiated, it could quickly produce gas and water. In the finite aquifer Experiment 3-1, after the water production of drainage Well 2 increased sharply during the initial stage of the well startup, the aquifer energy was gradually depleted. Meanwhile, the ${\text{Q}}_{{\text{w}}}$ gradually decreased to only 0.07 mL/min in the later stage. In infinite aquifer Experiment 4-1, since the aquifer’s energy in the production process was not depleted, the water production of drainage Well 2 rapidly increased and then stabilized at approximately 5 mL/min, indicating that the water invasion rate and the drainage rate reached an equilibrium state. Thereafter, Well 2 continued to produce water at this rate until the end of production. The average ${\text{Q}}_{{\text{w}}}$ from the infinite aquifer was 5.1 mL/min, which was approximately 12 times of that from the finite aquifer. The ${\text{W}}_{{\text{p}}}$ was as high as 721.7 mL, which was 9.2 times that of the infinite aquifer.

In the experiments, drainage Well 2 could achieve rapid and large-scale drainage. After the energy of the aquifer in the gas reservoir was released, it would not intrude toward the direction of Well 1. By the end of production, Well 1 in the finite aquifer Experiment 3-1 produced almost no water, and the infinite aquifer Experiment 4-1 also produced only 14.4 mL of water.

#### Dynamic pressure drop

**Figure 21:**
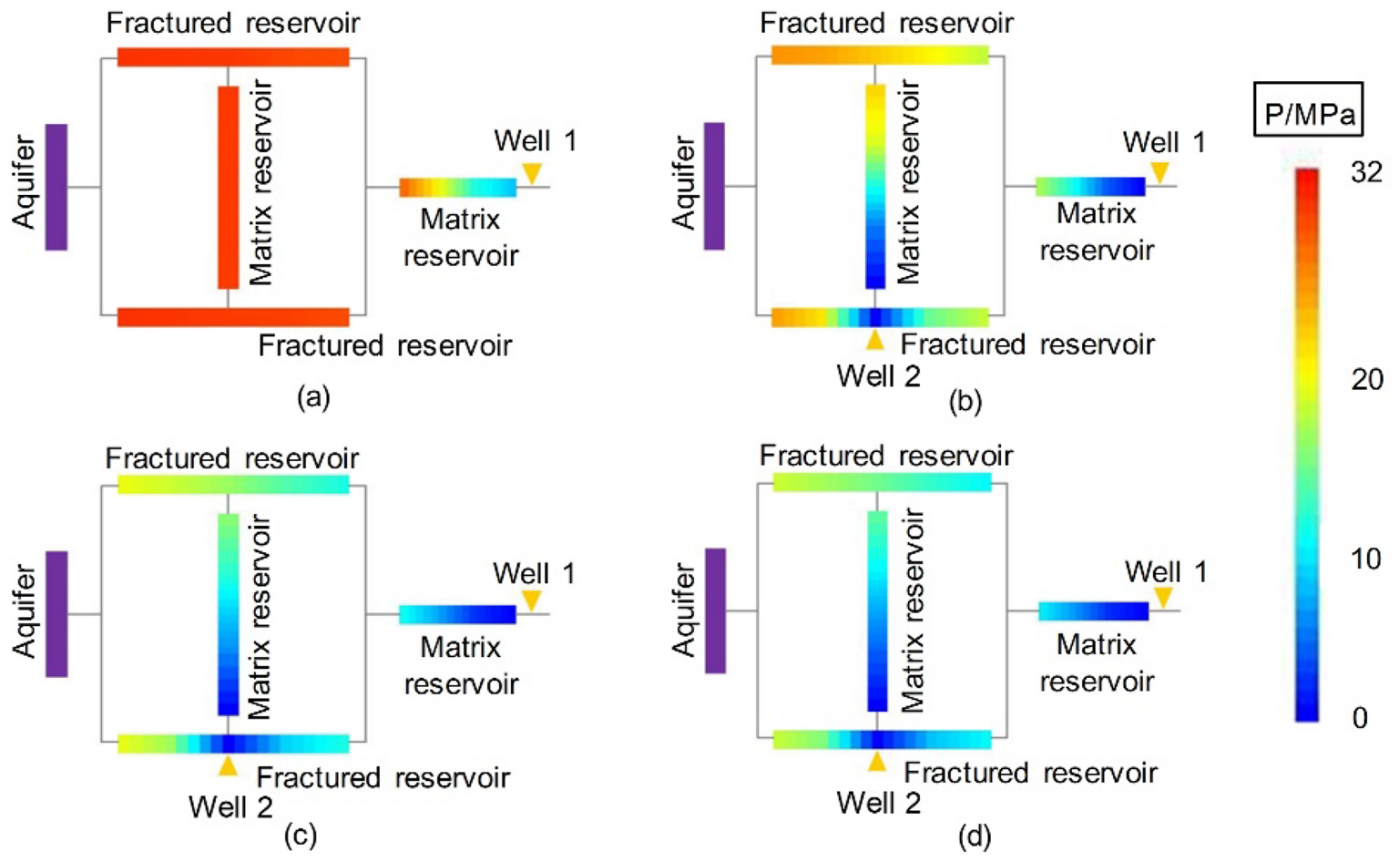
Pressure distributions at different stages of Experiment 4-1 (Model 2, infinite aquifer): (**a**) stable production of Well 1 ended -23 min, (**b**) end of the stabilized period of Well 2—45 min, (**c**) late production period -70 min, and (**d**) gas well production halted—142 min.

Figure 21 shows the dynamic pressure profiles of Experiment 4-1 (Geometrical Model 2, infinite aquifer). From the early stage of production of Well 1 until the stable production of the well ended, the reserve development was mainly concentrated in the immediate vicinity of Well 1. The pressure remained almost unchanged due to water invasion on the left side, far from the well area (Fig. 21a). Well 2 produced a large amount of water, forming a pressure drop funnel around the bottom of LFZ 2 and the connected MZ 1. As production progressed, the pressure drop funnel around Well 2 gradually expanded outward, allowing the undeveloped reserves of the area far from Well 1 (Cores 1-3) to be developed. Due to the infinite volume of the aquifer in this case, the residual pressure of the formation remained high until the gas well was shut in.

Comparing the dynamic pressure drops of Experiments 3-1 and 4-1 (Figs. 18 and 21), it can be seen that the aquifer size affects the ${\text{R}}$ of the entire gas reservoir. At the end of the stable production of Well 1 in the first stage of Experiments 3-1 and 4-1, the pressure drop funnels in the near-well area (Core 4) were 0.75 MPa and 1.14 MPa, respectively, and the pressure gradient of the infinite aquifer was clearly higher. Until the end of the water drainage process, the average residual pressure of the infinite aquifer case remained higher than that of the finite aquifer case (Figs. 18d and 21d).

#### Distribution of residual water and reserves

After water drainage was conducted with the newly added Well 2 in Experiments 3-1 and 4-1, the water saturations of the gas reservoirs were similar, at 31.22% and 34.01%, respectively. The difference is mainly due to LFZ 2, where drainage Well 2 was located. In Experiment 4-1, the water content in the area was as high as 46.51%, which was 7.65% higher than the same area of Experiment 3-1, indicating that the large fractures were the main channels of the water invasion (Table 13).

**Table 13:** Water saturation increment (%) in different zones at the ends of Experiments 3-1 and 4-1.

Experimental No	SFZ 1—Core 1	MZ 1—Core 2	LFZ 2—Core 3	MZ 2—Core 4	Average in the gas reservoir
3-1	37.42	19.74	38.86	28.87	31.22
4-1	35.16	19.67	46.51	34.68	34.01

The proportions of the residual reserves in different zones of the gas reservoir after gas production in the two types of aquifers of Experiments 3-1 and 4-1 are shown in Table 14.

**Table 14:** Distribution of residual reserves (%) in different zones at the ends of Experiments 3-1 and 4-1.

Experimental No	SFZ 1—Core 1	MZ 1—Core 2	LFZ 2—Core 3	MZ 2—Core 4
3-1	41.07	23.08	26.95	8.90
4-1	43.48	21.93	26.64	7.95

In Experiments 3-1 and 4-1, compared with other zones, MZ 2 had the highest ${\text{R}}$ of the reservoir; conversely, SFZ 1 had the lowest ${\text{R}}$ in the reservoir. Although MZ 2 had poor physical properties and low permeability, it was farthest from the aquifer but nearest to Well 1, so it had the highest ${\text{R}}$ of the gas reservoir. However, although SFZ 1 had favorable physical properties, it had the worst ${\text{R}}$ because it was directly connected to the aquifer and was the farthest from Well 1 and Well 2.

## Conclusions

The following conclusions were drawn according to the above-mentioned work:

1. When the fracture scale is appropriate and the permeability of a matrix zone is low, a production well should be deployed in an area that is close to the fractured zone. Although the fractured zone may lead to a fast water breakthrough, it will also result in a higher capacity for gas production and draining water, which can effectively avoid water invasion from occurring in the low-permeability zone.
2. In different zones of a gas reservoir, the water invasion mechanisms will be different. In a matrix area surrounded by fractures, the water saturation is considerably lower than that in the peripheral fractured zones after production. It is confirmed that the main reason for the water invasion in the matrix area is the imbibition caused by capillary forces.
3. The function of the drainage well varies with its location. A shorter distance between the drainage well location and the aquifer induces a higher drainage capacity and a lower gas production capacity.
4. In the process of development, a gas reservoir will be seriously flooded due to untimely drainage. If the remaining energy of this type of reservoir is insufficient, it is difficult to break the gas reservoir seal, even if more gas wells are drilled later.
5. The larger an aquifer is, the lower its ${\text{R}}$, and the larger the water drainage required to maintain gas production, resulting in a lower production benefit.

## Supplementary information

Supplementary information.

## List of symbols

${\text{A}}_{{\text{k}}}$: Core cross-sectional area measured at each measuring point, ${\text{L}}^{2}$
${\text{B}}_{{\text{W}}}$: Formation water volume coefficient
${\text{B}}_{{{\text{gi}}}}$: Original natural gas volumetric coefficient
${\text{G}}$: Natural gas reserves, ${\text{L}}^{3}$
${\text{G}}_{{\text{p}}}$: Cumulative gas production, ${\text{L}}^{3}$
${\text{L}}_{{\text{k}}}$: Core length measured by each measuring point, ${\text{L}}$
${\text{P}}$: Current formation pressure, ${\text{m}}/{\text{Lt}}^{2}$
${\text{P}}_{{\text{i}}}$: Initial reservoir pressure, ${\text{m}}/{\text{Lt}}^{2}$
${\text{P}}_{{\text{k}}}$: Average formation pressure of the core measured at each measuring point, ${\text{m}}/{\text{Lt}}^{2}$
${\text{P}}_{{{\text{rk}}}}$: Residual pressure in the cores measured at each measuring point, ${\text{m}}/{\text{Lt}}^{2}$
${\overline{\text{P}}}$: Average formation pressure of the gas reservoir, ${\text{m}}/{\text{Lt}}^{2}$
$${\text{R}}$$: Recovery factor
${\text{S}}_{{{\text{gk}}}}$: Residual gas saturation measured in the core
${\text{W}}_{{\text{e}}}$: Cumulative water influx, ${\text{L}}^{3}$
${\text{W}}_{{\text{p}}}$: Cumulative water production, ${\text{L}}^{3}$
${\text{Z}}$: Natural gas deviation coefficient at pressure *P*
${\text{Z}}_{{\text{i}}}$: Natural gas deviation coefficient at pressure ${\text{P}}_{{\text{i}}}$
${\uptheta }$: Relative apparent pressure of formation
${\upomega }$: Water invasion volume coefficient
$\emptyset_{{\text{k}}}$: Core porosity measured at each measuring point

## Subscripts

$k$: Property at each measuring point
$$i$$: Initial condition

## References

[1] Beattie, D. R., & Roberts, B. E. Water coning in naturally fractured gas reservoirs. Presented at the SPE Gas Technology Symposium, Calgary, Alberta, 28 April-1 May. SPE-35643-MS. 10.2118/35643-MS (1996).

[2] Liu HX, Ren D, Gao SS (2015). Water influx mechanism and development strategy of gas reservoirs with edge and bottom water. Nat. Gas Industry..

[3] Hernandez, J. C., Wojtanowicz, A. K. & White, C. D. Effect of anisotropy on water invasion in edge-water drive reservoirs. Presented at the Canadian International Petroleum Conference, Calgary, Alberta, 13–15 June. PETSOC-2006–199. 10.2118/2006-199 (2006).

[4] Dotoku, S., Hutagalung, D. R. A., Purba, V. S. *et al.* Determining trapped gas saturation and effect on recovery factor in carbonate reservoir. Presented at the SPE Gas & Oil Technology Showcase and Conference, Dubai, UAE, 21–23 October. SPE-198556-MS. 10.2118/198556-MS (2019).

[5] Agarwal RG, Al-Hussainy R, Ramey HJ (1965). The importance of water influx in gas reservoirs. J. Pet. Technol..

[6] Farah, N., Ding, D. Y. & Wu, Y. S. Simulation of the impact of fracturing-fluid-induced formation damage in shale gas reservoirs. Presented at the SPE Reservoir Simulation Symposium, Houston, Texas, 23–25 February. SPE-173264-MS. 10.2118/173264-PA (2017).

[7] Li S, Pan Y, Sun L (2011). Thoughts and suggestions for the improvement of the efficiency and development level of complex gas fields. Nat. Gas Industry..

[8] Hearn, W. J. Gas well deliquification application overview. Presented at the Abu Dhabi International Petroleum Exhibition and Conference, Abu Dhabi, UAE, 1–4 November. SPE-138672-MS. 10.2118/138672-MS (2010).

[9] Yang, G., Honglan, Z., He, L. *et al.* Technique of water control and oil recovery based on water plugging combined with fracturing in low permeability and high water cut oilfield. Presented at the SPE Asia Pacific Oil and Gas Conference and Exhibition, Jakarta, Indonesia, 22–24 October. SPE-165863-MS. 10.2118/165863-MS (2013).

[10] Mattey, P., Varshney, M., Daksh, P. V. *et al.* A successful water shut off treatment in gas well of bassein field by polymeric gel system. Presented at the SPE Oil and Gas India Conference and Exhibition, Mumbai, India, 4–6 April. SPE-185371-MS. 10.2118/185371-MS (2017).

[11] Ghosh B, Ali SA, Belhaj H (2020). Controlling excess water production in fractured carbonate reservoirs: Chemical zonal protection design. J. Pet. Explor. Prod. Technol..

[12] Feng X, Zhong B, Yang XF (2015). Effective water influx control in gas reservoir development: Problems and countermeasures. Nat. Gas Industry..

[13] Warren JE, Root PJ (1963). The behavior of naturally fractured reservoirs. SPE J..

[14] Xia CS (2002). Ways and methods of enhancing recovery in various water-carrying gas reservoirs. Nat. Gas Industry..

[15] Jin, L., & Wojtanowicz, A. K. Coning control and recovery improvement using in-situ water drainage/injection in bottom/water/drive reservoir. Presented at the SPE Improved Oil Recovery Symposium, Tulsa, Oklahoma, 24–28 April. SPE-129663-MS. doi: 10.2118/129663-MS (2010).

[16] Kabir, C. S., Parekh, B. & Mustafa M. A. Material-balance analysis of gas reservoirs with diverse drive mechanisms. Presented at the SPE Annual Technical Conference and Exhibition, Houston, Texas, 28–30 September. SPE-175005-MS. 10.2118/175005-MS (2015).

[17] Glumov, D. N., Sokolov, S. V. & Strekalov, A. V. Assessment of drained gas reserves in the process of gas and gas condensate field operation in water drive. Presented at the SPE Russian Petroleum Technology Conference, Moscow, Russia. 16–18 October. SPE-187863-MS. 10.2118/187863-MS (2017)

[18] Lakatos, I. J., Bodi, T. & Lakatos-Szabo, J. Water induced formation damage in unconventional gas reservoirs. Presented at the 8th European Formation Damage Conference, Scheveningen, The Netherlands, 27–29 May. SPE-121944-MS. 10.2118/121944-MS (2009).

[19] Li, K. & Zhang, H. Experimental study of water shut-off by wettability alteration to gas wetness. Presented at the SPE EUROPEC/EAGE Annual Conference and Exhibition, Vienna, Austria, 23–26 May. SPE-143483-MS. 10.2118/143483-MS (2011).

[20] Xu X, Wan YJ, Chen YL (2019). Physical simulation of water invasion and water control for the fractured water-bearing gas reservoirs. Nat. Gas Geosci..

[21] Fang FF, Shen WJ, Li XZ (2019). Experimental study on water invasion mechanism of fractured carbonate gas reservoirs in Longwangmiao Formation, Moxi block, Sichuan Basin. Environ. Earth Sci..

[22] Rezaee, M., Rostami, B., Zadeh, M. *et al.* Experimental determination of optimised production rate and its upscaling analysis in strong water drive gas reservoirs. Presented at the International Petroleum Technology Conference, Beijing, China, 26–28 March. IPTC-16938-Abstract. 10.2523/IPTC-16938-Abstract (2013).

[23] Liu SZ, Sun AY, Huang BG (1999). The prediction method of water influx and formation pressure for a water drive gas reservoir. Pet. Explor. Dev..

[24] Chen YF, He W, Luo T (1999). Research on water control method of water producing gas well with fracture water channeling. Nat. Gas Ind..

[25] Yang JH, Shao Y, Luo YP (2003). Effictiveness analysis of drainage water in Well Chi 27 and resolvent of boundary water invasion in carboniferous reservoirs in East SiChuan. Drill. Prod. Technol..

[26] Hao, C. L.. Study on water invasion characteristics and optimal water control measures of typical carboniferous gas reservoirs in Eastern Sichuan. MS thesis, Southwest Petroleum University, Sichuan, China, December 2014 (2014).

[27] Gou WN, Ran H, Li CH (2002). On-the-Spot testing and result analysis of early regulating water invasions in carboniferous gas reservoirs in Dachiganjing field. Nat. Gas Ind..

[28] Geertsma J, Croes GA, Schwarz N (1956). Theory of dimensionally scaled models of petroleum reservoirs. Trans. AIME.

[29] Jiao CY, Liu HX, Liu PF (2019). Similarity criterion of the physical simulating experiment for the development performances of low-permeability tight gas reservoirs. Pet. Geol. Oilfield Dev. Daqing..

[30] Chen YQ (1978). Judgment method of natural water invasion in gas field. Pet. Explor. Dev..

[31] Abdul-Majeed, G. H. & Al-Assal, J. R. Graphical method for estimating original gas in-place in water drive gas reservoirs. Society of Petroleum Engineers. SPE-15840-MS (1986).

[32] Siddiqui, F., Waqas, G. M. & Khan, M. N. Application of general material balance on gas condensate reservoirs GIIP estimation. Presented at the SPE/PAPG Annual Technical Conference, Islamabad, Pakistan, 10–11 November. SPE-142847-MS. 10.2118/142847-MS (2010).

[33] Xu G, Yin H, Yuan H (2020). Decline curve analysis for multiple-fractured horizontal wells in tight oil reservoirs. Adv. Geo-Energy Res..

[34] Xu X, Liu XW, Yang ZM (2012). An experimental study on single-phase seepage characteristics with a large-scale model made of ultra-low permeability sandstone outcrops. Acta Pet. Sinica..

[35] Feng X, Yang XF, Deng H (2013). Identification of the water invasion law in high-sulfur and edge-water gas reservoirs based on the characteristics of pressure variation in the water zone. Nat. Gas Ind..

[36] Özkaya SI (2019). Fracture modeling from borehole image logs and water invasion in carbonate reservoirs with layer-bound fractures and fracture corridors. J. Pet. Sci. Eng..

