# Supplementary material for: Physical simulation for water invasion and water control optimization in water drive gas reservoirs
Source: Sci Rep. 2021 Mar 18;11:6301. doi: 10.1038/s41598-021-85548-0 (PMC7973525; doi:10.1038/s41598-021-85548-0)
Supplement: Supplementary file 1 — Supplementary information. [file 41598_2021_85548_MOESM1_ESM.docx]

**Supplementary files**

**Paper Title:** Physical Simulation for Water Invasion and Water Control Optimization in Water Drive Gas Reservoirs

**Author(s):** Xuan Xu, Xizhe Li, Yong Hu, Qingyan Mei , Yu Shi, Chunyan Jiao

**Appendix A—Detail of the Water Invasion Volume Coefficient Method**

Currently, the main methods of water invasion degree identification were developed based on the gas reservoir material balance equation, so generally, the evaluation results of the three methods for the same production data are quite similar, as Siddiqui et al. noted in their work on the application of the general material balance equation^1-3^.

Two parameters, namely, the gas recovery and the relative apparent pressure of the formation, are utilized in the water invasion volume coefficient method, which is equivalent to the normalized pressure drop curve method. The water invasion volume coefficient method is more suitable for a comparative analysis of water invasion with different gas reserves and formation pressures. However, the water influx volume coefficient method requires more gas reservoir parameters, such as the original gas reserves, compared to the other two methods. As such, the water invasion volume coefficient method was utilized to analyze the water invasion and development performance of the gas reservoir in the experiments.

The water invasion volume coefficient is defined as the ratio of the net water amount invading into the gas-bearing area to the primary reserves of the gas reservoir, namely,

$\text{ω=}\frac{\text{W}_{\text{e}}\text{-}\text{W}_{\text{p}}\text{B}_{\text{W}}}{\text{G}\text{B}_{\text{gi}}}$. …………………………………………………………..……..………… (A-1)

The pressure drop equation of a normal pressure WDGR can also be expressed as

$\frac{\text{P}}{\text{Z}}\text{=}\frac{\text{P}_{\text{i}}}{\text{Z}_{\text{i}}}\text{(}\frac{\text{G-}\text{G}_{\text{p}}}{\text{G-}\text{ωG}}\text{)}$. ……………….…………………………….…………….………………....(A-2)

The $\text{R}$ of reserves is defined as

$\text{R=}\frac{G_{p}}{G}$. ………………………….………………………………...…………………..…...(A-3)

The relative apparent pressure of formation is defined as

$\text{θ=}\frac{\text{P/Z}}{\text{P}_{\text{i}}\text{/}\text{Z}_{\text{i}}}$. ………………………………….……………………………………………....(A-4)

From the above relationship,

$\text{θ=}\frac{\text{1}\text{-R}}{\text{1}\text{-ω}}$. …………………………….……………………………………………….……(A-5)

For non-water drive gas reservoirs, $\omega=0$, and Eq. (5) becomes

$\text{θ=}\text{1}\text{-R}$. ………………………………….………………………………………………...(A-6)

Eq. (5) shows that the angle between the $\text{θ\textasciitilde R}$ curve and the longitudinal axis is greater than 45° for WDGRs since the water invasion volume coefficient $\omega<1$. In contrast, for volumetric gas reservoirs, the angle between the $\text{θ\textasciitilde R}$ curve and the longitudinal axis is 45°.

It is difficult to obtain an accurate average formation pressure during actual production. In the experiments, the internal pressures of the gas reservoir are accurately measured in real time at more than 20 measurement points, which provides a sound foundation for accurately obtaining the average formation pressure of the entire gas reservoir. This is beneficial to analyzing the degree of water invasion in the gas reservoir.

According to the experiments, the pressure and physical reservoir parameters of the different zones of the gas reservoir are different, so the average formation pressure of the gas reservoir is calculated by the weighted average values of the physical parameters related to the reserves:

$\bar{\text{P}}\text{=}\frac{\sum_{\text{k=1}}^{\text{n}} \text{p}_{\text{k}}{\text{L}_{\text{k}}\text{A}_{\text{k}}\text{∅}}_{\text{k}}}{\sum_{\text{k=1}}^{\text{n}} {\text{L}_{\text{k}}\text{A}_{\text{k}}\text{∅}}_{\text{k}}}$. …………………………….………………….……………………..….…(A-7)

At the same time, due to the large pressure variation range in the gas reservoir during the gas reservoir development process, the value of the nitrogen deviation factor under different pressures should be considered when calculating the apparent formation pressure $\text{P/Z}$.

Compared with the pressure drawdown curve method, the water invasion volume coefficient method requires a more accurate and reliable estimation of the initial in-place reserves of the gas reservoirs, in addition to accurate average formation pressure, to calculate the $\text{R}$ of the gas reservoirs, posing additional challenges for gas reservoir developers. In the experiment, the reservoir pore volume and the initial reservoir pressure are already known, so the primary reserves can be accurately obtained.

**References**

1. 30. Chen, Y. Q. 1978. Judgment Method of Natural Water Invasion in Gas Field. Pet. Exploration and Development. 5(3): 51-57. <https://doi.org/CNKI:SUN:SKYK.0.1978-03-005>.
2. 31. Abdul-Majeed, G. H. and Al-Assal, J. R. 1986. Graphical Method for Estimating Original Gas In-Place in Water Drive Gas Reservoirs. Society of Petroleum Engineers. SPE-15840-MS. <https://doi.org/NA>.
3. 32. Siddiqui, F., Waqas, G. M. and Khan, M. N. 2010. Application of General Material Balance on Gas Condensate Reservoirs GIIP Estimation. Presented at the SPE/PAPG Annual Technical Conference, Islamabad, Pakistan, 10-11 November. SPE-142847-MS. https://doi.org/10.2118/142847-MS.
